# Annotation of Protein Domains Reveals Remarkable Conservation in the Functional Make up of Proteomes Across Superkingdoms

**DOI:** 10.3390/genes2040869

**Published:** 2011-11-08

**Authors:** Arshan Nasir, Aisha Naeem, Muhammad Jawad Khan, Horacio D. Lopez-Nicora, Gustavo Caetano-Anollés

**Affiliations:** 1 Evolutionary Bioinformatics Laboratory, Department of Crop Sciences, University of Illinois, Urbana, IL 61801, USA; E-Mail: anasir@illinois.edu; 2 Mammalian NutriPhysioGenomics Laboratory, Department of Animal Sciences, University of Illinois, Urbana, IL 61801, USA; E-Mails: naeem1@illinois.edu (A.Na.); khan41@illinois.edu (M.J.K.); 3 Plant Pathology Laboratory, Department of Crop Sciences, University of Illinois, Urbana, IL 61801, USA; E-Mail: hlopezn2@illinois.edu

**Keywords:** functional annotation, fold superfamily, molecular function, protein domain, SCOP, structure, superkingdom

## Abstract

The functional repertoire of a cell is largely embodied in its proteome, the collection of proteins encoded in the genome of an organism. The molecular functions of proteins are the direct consequence of their structure and structure can be inferred from sequence using hidden Markov models of structural recognition. Here we analyze the functional annotation of protein domain structures in almost a thousand sequenced genomes, exploring the functional and structural diversity of proteomes. We find there is a remarkable conservation in the distribution of domains with respect to the molecular functions they perform in the three superkingdoms of life. In general, most of the protein repertoire is spent in functions related to metabolic processes but there are significant differences in the usage of domains for regulatory and extra-cellular processes both within and between superkingdoms. Our results support the hypotheses that the proteomes of superkingdom Eukarya evolved via genome expansion mechanisms that were directed towards innovating new domain architectures for regulatory and extra/intracellular process functions needed for example to maintain the integrity of multicellular structure or to interact with environmental biotic and abiotic factors (e.g., cell signaling and adhesion, immune responses, and toxin production). Proteomes of microbial superkingdoms Archaea and Bacteria retained fewer numbers of domains and maintained simple and smaller protein repertoires. Viruses appear to play an important role in the evolution of superkingdoms. We finally identify few genomic outliers that deviate significantly from the conserved functional design. These include *Nanoarchaeum equitans*, proteobacterial symbionts of insects with extremely reduced genomes, Tenericutes and *Guillardia theta*. These organisms spend most of their domains on information functions, including translation and transcription, rather than on metabolism and harbor a domain repertoire characteristic of parasitic organisms. In contrast, the functional repertoire of the proteomes of the Planctomycetes-Verrucomicrobia-Chlamydiae superphylum was no different than the rest of bacteria, failing to support claims of them representing a separate superkingdom. In turn, Protista and Bacteria shared similar functional distribution patterns suggesting an ancestral evolutionary link between these groups.

## 1. Introduction

Proteins are active components of molecular machinery that perform vital functions for cellular and organismal life [1,2]. Information in the DNA is copied into messenger RNA that is generally translated into proteins by the ribosome. Nascent polypeptide chains are unfolded random coils but quickly undergo conformational changes to produce characteristic and functional folds. These folds are three-dimensional (3D) structures that define the native state of proteins [3,4]. Biologically active proteins are made up of well-packed structural and functional units referred to as domains. Domains appear either singly or in combination with other domains in a protein and act as modules by engaging in combinatorial interplays that enhance the functional repertoires of cells [5]. While molecular interactions between domains in mutidomain proteins play important roles in the evolution of protein repertoires [6], it is the domain structure that is maintained in proteins for long periods of evolutionary time [7–9]. This is in sharp contrast to amino acid sequence, which is highly variable. For this reason, protein domains are also considered evolutionary units [7,10–12].

### 1.1. Classification of Domains

Domains that are evolutionarily related can be grouped together in hierarchical classifications [1,10,13]. One scheme of classifying protein domains is the well-established “Structural Classification of Proteins” (SCOP). The SCOP database groups domains that have sequence conservation (generally with >30% pairwise amino acid residue identities) into fold families (FFs), FFs with structural and functional evidence of common ancestry into fold superfamilies (FSFs), FSFs with common 3D structural topologies into folds (Fs), and Fs sharing a same general architecture into protein classes [10,14]. SCOP identifies protein domains using concise classification strings (css) (e.g., c.26.1.2, where c represents the protein class, 26 the F, 1 the FSF and 2 the FF). The 97,178 domains indexed in SCOP 1.73 (corresponding to 34,494 PDB entries) are classified into 1,086 F, 1,777 FSFs, and 3,464 FFs. Compared to the number of protein entries in UniProt (531,473 total entries as of July 27, 2011) the number of domain structural designs at these different levels of structural abstraction is quite limited. Their relatively small number suggests that fold space is finite and is evolutionarily highly conserved [1,7,15].

### 1.2. Assigning FSF Structures to Proteomes

Genome-encoded proteins can be scanned against advanced linear hidden Markov models (HMMs) of structural recognition in SUPERFAMILY [16,17]. HMM libraries are generated using the iterative Sequence Alignment and Modeling (SAM) method. SAM is considered one of the most powerful algorithms for detecting remote homologies [18]. The SUPERFAMILY database currently provides FSF structural assignments for a total of 1,245 model organisms including 96 Archaea, 861 Bacteria and 288 Eukarya.

### 1.3. Assigning Functional Categories to Protein Domains

Assigning molecular functions to FSFs is a difficult task since approximately 80% of the FSFs defined in SCOP are multi-functional and highly diverse [19]. For example, most of the ancient FSFs, such as the P-loop-containing NTP hydrolase FSF (c.37.1), are highly abundant in nature and include many FFs (20 in case of c.37.1). Each of those families may have functions that impinge on multiple and distinct pathways or networks. The functional annotation scheme introduced by Vogel and Chothia in SUPERFAMILY is a one-to-one mapping scheme that is based on information from various resources, including the Cluster of Orthologus Groups (COG) and Gene Ontology (GO) databases and manual surveys [20–23]. When a FSF is involved in multiple functions, the most predominant function is assigned to that multi-functional FSF under the assumption that the most dominant function is the most ancient and predominantly present in all proteomes. The error rate in assignments is estimated to be <10% for large FSFs and <20% for all FSFs [23].

The SUPERFAMILY functional classification maps seven general functional categories to 50 detailed functional categories in a two-tier hierarchy (Table 1). The seven general categories include *Metabolism*, *Information*, *Intracellular processes* (*ICP*), *Extracellular processes* (*ECP*), *Regulation*, *General*, and *Other* (we will refer to them as “categories” and “functional repertoires” interchangeably). In this study, we take advantage of this coarse-grained functional annotation scheme to assign individual functional categories to FSFs. We are aware that this one-to-one mapping may not provide a complete profile for multi-functional domains [19]. Dissection of such detailed functions and their comparison across organisms is a difficult problem that we will not address in this study. In contrast, we focus on domains defined at FSF level and use the coarse-grained functional annotation scheme to explore the functional diversity of the proteomes encoded in genomes that have been completely sequenced. Our results yield a global picture of the functional organization of proteomes that is only possible with this classification scheme. Results suggest that the functional structure of proteomes is remarkably conserved across all organisms, ranging from small bacteria to complex eukaryotes. There is also evidence for the existence of few outliers that deviate from global trends. Here we explore what makes these proteomes distinct.

## 2. Results and Discussion

### 2.1. General Patterns in the Distribution of FSF Domain Functions

**Figure 1:**
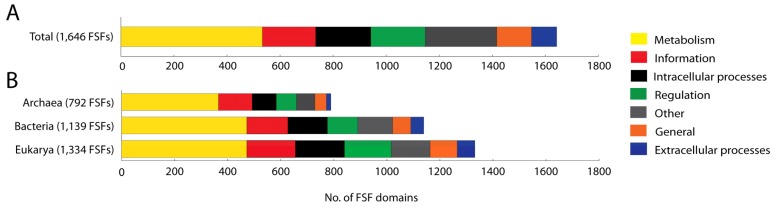
Number of protein FSFs annotated for each functional category defined in SCOP 1.73 (**A**) and in the three superkingdoms (**B**). The functional distributions show that coarse-grained functions are conserved across cellular proteomes and *Metabolism* is the most dominant functional category. Numbers in parentheses indicate the total number of FSFs annotated in each dataset. The number of FSFs increases in the order Archaea, Bacteria and Eukarya.

We studied the molecular functions of 1,646 domains defined at the FSF level of structural abstraction (SCOP 1.73) that are present in the proteomes of a total of 965 organisms spanning the three superkingdoms. A total of 135 FSFs that could not be annotated were excluded from analysis. For these FSFs, the functional annotation is not available. Out of the 1,646 FSFs studied, approximately one-third (32.38%) performs molecular functions related to *Metabolism*. Categories *Other* (16.58%), *ICP* (12.63%), *Regulation* (12.45%), and *Information* (12.21%) are uniformly distributed within proteomes. In contrast, *General* (7.96%) and *ECP* (5.77%) are significantly underrepresented compared to the rest (Figure 1(A)). The total number of FSFs in each category exhibits the following decreasing trend: *Metabolism > Other > ICP > Regulation > Information > General > ECP*. These patterns of FSF number and relative proteome content are for the most part maintained when studying the functional annotation of FSFs belonging to each superkingdom (Figure 1(B)). However, the number of FSFs in each superkingdom varies considerably and increases in the order Archaea, Bacteria and Eukarya, as we have shown in earlier studies [7].

The significantly higher number of FSFs devoted to *Metabolism* is an anticipated result given the central importance of metabolic networks. However, the much larger number of FSFs corresponding to *Other* is quite unexpected. The 273 FSFs belonging to this category include 200 and 73 FSFs in sub-categories *unknown functions* and *viral proteins*, respectively. The sub-category *unknown function* includes FSFs for which the functions are either unknown or are unclassifiable. Viruses are defined as simple biological entities that are considered to be “gene poor” relatives of cellular organisms [24]. However, the number of domains belonging to *viral proteins* that are present in cellular organisms makes a noteworthy contribution to the total pool of FSFs (4.43%). Thus, viruses have a much more rich and diverse repertoire of domain structures than previously thought and their association with cellular life has contributed considerable structural diversity to the proteomic make up (A. Nasir, K.M. Kim and G. Caetano-Anollés, ms. in preparation).

The numbers of FSFs belonging to categories *Regulation*, *Information*, and *ICP* are uniformly distributed in proteomes. However, the *ECP* category is the least represented, perhaps because this category is the last to appear in evolution [7,15]. Extra cellular processes are more important to multicellular organisms (mainly eukaryotes) than to unicellular organisms. Multicellular organisms need efficient communication, such as signaling and cell adhesion. They also trigger immune responses and produce toxins when defending from parasites and pathogens. These *ECP* processes, which are depicted in the minor categories of *cell adhesion*, *immune response*, *blood clotting* and *toxins/defense*, are needed when interacting with environmental biotic and abiotic factors and for maintaining the integrity of multicellular structure. These categories are also present in the microbial superkingdoms but their functional role may be different than in Eukarya.

We note that current genomic research is highly shifted towards the sequencing of microbial genomes, especially those that hold parasitic lifestyles and are of bacterial origin. In fact, 67% of proteomes in our dataset belong to Bacteria. This bias can affect conclusions drawn from global trends such as those in Figure 1(A), including the under-representation of *ECP* FFs, because of their decreased representation in microbial proteomes.

### 2.2. Distribution of FSF Domain Functions in the Three Superkingdoms of Life

**Figure 2:**
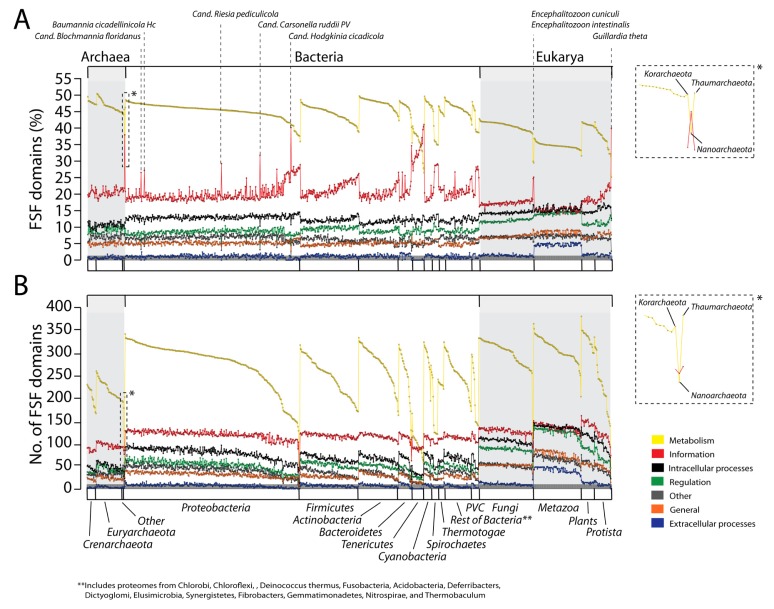
The functional distribution of FSFs in individual proteomes of the three superkingdoms. Both the percentage (**A**) and actual FSF numbers (**B**) indicate conservation of functional distributions in proteomes and the existence of considerable functional flexibility between superkingdoms. Dotted vertical lines indicate genomic outliers. Insets highlight the interplay between *Metabolism* (yellow trend lines) and *Information* (red trend lines) in *N. equitans*.

In order to explore whether the overall distribution of general functional categories differs in organisms belonging to the three superkingdoms, we analyzed proteomes at the species level and calculated both the percentage and actual number of FSFs corresponding to different functional repertoires (Figure 2).

FSF domains follow the following decreasing trend in both the percentage and actual counts of FSFs, and do so consistently for the three superkingdoms: *Metabolism > Information > ICP > Regulation > Other > General > ECP*. Note that trend lines across proteomes seldom overlap and cross in Figure 2. It is noteworthy however that this trend differs from the decreasing total numbers of FSFs we described above (Figure 1). Thus, no correlation should be expected between the numbers of FSFs for individual proteomes and the total set for each category. This suggests that variation in functional assignments across proteomes of superkingdoms may not necessarily match overall functional patterns.

Proteomes in microbial superkingdoms Archaea and Bacteria exhibit remarkably similar functional distributions of FSFs (Figure 2(A)). The only exception appears to be the slight overrepresentation of *Regulation* FSFs (green trend lines) and underrepresentation of *ICP* (black trend lines) in Archaea compared to Bacteria (especially Proteobacteria). These distributions are clearly distinct from those in Eukarya. Proteomic representations of FSFs corresponding to *Metabolism* and *Information* are decreased while those of all other five functional categories are significantly and consistently increased (Figure 2(A)). There is also more variation evident in Eukarya; large groups of proteomes exhibit different patterns of functional use (clearly evident in *Information*; red trend lines in Figure 2(A)).

On the whole, the relative functional make up of the proteomes of individual superkingdoms appear highly conserved (Figure 2(A)). There is however considerable variation in the metabolic functional repertoire of organisms, especially in Bacteria, where *Metabolism* ranges 30–50% of proteomic content (100–350 FSFs, Table S1 and Table S2). This variation is not present in other functional repertoires.

Consequently, tendencies of reduction in the metabolic repertoire are generally offset by small increases in the representation of the other six repertoires, with the notable exception of *Information*. In this particular case, when *Metabolism* goes down *Information* goes up. For example, bacterial proteomes with metabolic FSF repertoires of <45% offset their decrease by a corresponding increase in *Information* FSFs (generally from ∼20% to ∼35%, Figure 2(A)). In all superkingdoms, we identify groups of proteomes or few outliers that deviate from the global trends (vertical dotted lines in Figure 2(A)). As we will discuss below this is generally a consequence of reductive evolution imposed by the lifestyle of organisms (discussed in detail below). Outliers are particularly evident in Bacteria and harbor sharp increases in *Information* repertoires, not always with corresponding decreases in *Metabolism*. In Archaea, decreases of *Metabolism* are generally offset by increases of the *Regulation* category, with an exception in *Nanoarchaeum equitans* (see below). In Eukarya, decreases in *Metabolism* go in hand with decreases in *Information*, and are correspondingly offset mostly by increases in *Regulation* and *ECP*. Apparently, the advantages of regulatory control (e.g., signal transduction and transcriptional and posttranscriptional regulation) and multicellularity counteract the interplay of *Metabolism* and *Information* in eukaryotes.

When we look at the actual number of FSFs within each functional repertoire (Figure 2(B)), we observe a clear trend in domain use that matches the total trend for superkingdoms described above (Figure 1). In most cases, the functional repertoires of Archaea are smaller than those of Bacteria, and bacterial repertoires are generally smaller than those of Eukarya (Figure 2(B)). This holds true for all functional categories. However, the numbers of metabolic FSFs vary 1.5–4 fold in proteomes of superkingdoms, the change being maximal in Bacteria. While both proteomes in Eukarya and Bacteria show similar ranges of metabolic FSFs, the repertoire of Archaea is more constrained. Furthermore, FSFs belonging to categories *Other* and *ECP* are significantly higher in Eukarya than in the microbial superkingdoms. These remarkable observations suggest high conservation in the make up of proteomes of superkingdoms and at the same time considerable levels of flexibility in the metabolic make-up of organisms. Results also support the evolution of the protein complements of Archaea and Bacteria via reductive evolutionary processes and Eukarya by genome expansion mechanisms [7,25]. Reductive tendencies in microbial superkingdoms do not show bias in favor of any functional category. Furthermore, enrichment of eukaryal proteomes with viral proteins supports theories, which state that viruses have played an important role in the evolution of Eukarya [26].

### 2.3. Distribution of FSF Domain Functions in Individual Phyla/Kingdoms

Figure 2 also describes the functional distribution of FSFs at the phyla/kingdom level for each superkingdom. Plots describing the percentages (Figure 2(A)) and actual number of FSFs in proteomes (Figure 2(B)) highlight the existence of “outliers” (vertical dotted lines in Figure 2(A)) that deviate from the global functional trends that are typical of each superkingdom.

In Archaea, the functional repertoires of the proteomes of Euryarachaeota, Crenarchaeota, Korarcheota and Thaumarchaeota were remarkably conserved and consistent with each other. Only *N. equitans* could be considered an outlier (insets of Figure 2). Its proteome deviates from the global archaeal signature by reducing its proteomic make up (it has only 200 distinct FSFs) and by exchanging *Information* for metabolic FSFs. *N. equitans* is an obligate intracellular parasite [27] that is part of a new phylum of Archaea, the Nanoarchaeota [28]. *N. equitans* has many atypical features, including the almost complete absence of operons and presence of split genes [29], tRNA genes that code for only half of the tRNA molecule [30], and the complete absence of the nucleic acid processing enzyme RNAse P [31]. Some of these features were used to propose that *N. equitans* is a living fossil [32], represents the root of superkingdom Archaea and the tree of life [33], and is part of a very ancient and yet to be described superkingdom (M. Di Giulio, personal communication). Phylogenomic analyses of domain structures in proteomes suggest Archaea is the most ancient superkingdom [19,34] and has placed *N. equitans* at the base of the tree of life together with other archaeal species. Its ancestral nature is therefore in line with the evolutionary and functional uniqueness of *N. equitans* and the very distinct functional repertoire we here report.

In Bacteria, the functional repertoires of bacterial phyla were also remarkably conserved. Only *Information* and *Metabolism* showed significantly distinct patterns and considerable variation in the use of FSFs. Again, decreases in representation of metabolic FSFs were generally offset by increases in informational FSFs (Figure 2(A)). Notable outliers include the Tenericutes and the Spirochetes. As groups, they have the highest relative usage of *Information* FSFs, which are clearly offset by a decrease in metabolic FSFs. The Tenericutes is a phylum of bacteria that includes class Mollicutes. Members of the Mollicutes are typical obligate parasites of animals and plants (some of medical significance such as *Mycoplasma*) that lack cell walls and have gliding motility. These organisms are characterized by small genome sizes [35] considered to have evolved via reductive evolutionary processes [36]. Because of its unique properties and history, mycoplasmas have been used recently to produce a completely synthetic genome [37]. There were also clear outliers in the Proteobacteria. These included Candidatus *Blochmannia floridanus* (symbiont of ants), *Baumannia cicadellinicola* (symbiont of sharpshooter insect), Candidatus *Riesia pediculicola,* Candidatus *Carsonella ruddii* (symbiont of sap-feeding insects) and Candidatus *Hodgkinia cicadicola* (symbiont of cicadas). These bacteria are generally endosymbionts of insects (e.g., ants, sharpshooters, psyllids, cicadas) that have undergone irreversible specialization to an intracellular lifestyle. Candidatus *Carsonella ruddii* has the smallest genome of any bacteria [38]. There were also bacterial proteome groups that were expected to be outliers but were no different than the rest. Bacteria belonging to the superphylum Planctomycetes-Verrucomicrobia-Chlamydiae (PVC) are different from other bacterial phyla because they have an “eukaryotic touch” [39]. Indeed, PVC bacteria display genetic and cellular features that are characteristics of Eukarya and Archaea, including the presence of Histone H1, condensed DNA surrounded by membrane, α-helical repeat domains and β-propeller folds that make up eukaryotic-like membrane coats, reproduction by budding, ether lipids and lack of cell walls [40–42]. Due to the unique nature of the PVC superphylum, it was proposed that these organisms be identified as a separate superkingdom that contributed to the evolution of Eukarya and Archaea [40]. However, trees of life generated from domain structures in hundreds of proteomes did not dissect the PVC superphylum into a separate group [7,19,34]. Functional distributions of FSFs now show PVC proteomes appear no different from bacteria (Figure 2). These results do not support PVC-inspired theories that explain the diversification of the three cellular superkingdoms of life.

In contrast to the functional repertoires of bacterial and archaeal phyla, proteomes belonging to individual kingdoms in Eukarya had functional signatures that were highly conserved (Figure 2(A)). However, these signatures differed between groups. Plants and fungi had functional representations that were very similar and showed little diversity. In contrast, Metazoa functional distributions increased the representation of *ECP* and *Regulation* FSFs in exchange of FSFs in *Metabolism* and *Information*. Protista had patterns that resemble those of Plants and Fungi but had widely varying metabolic repertoires, very much like Bacteria. This possible link between basal eukaryotes and bacteria revealed by our comparative analysis is consistent with the existence of an ancestor of Bacteria and Eukarya and the early rise of Archaea [34]. Only few outliers belonging to kingdoms Fungi (*Encephalitozoon cuniculi* and *Encephalitozoon intestinalis*) and Protista (*Guillardia theta*) were identified. *E. cuniculi* and *E. intestinalis* are eukaryotic parasites with highly reduced genomes [43,44]. Similarly, *Guillardia theta* is a nucleomorph that has a highly compact and reduced genome with loss of nearly all metabolic genes [45].

When we look at the actual number of FSFs in proteomes of phyla and kingdoms (Figure 2(B)) we observe that while the overall patterns match those of FSF representation (Figure 2(A)), FSF number revealed considerable variation in the metabolic repertoire of Protista and Bacteria. FSFs in these groups typically ranged 130–340, with PVC and Spirochetes exhibiting the smallest range (130–300 FSFs). In contrast, metabolic repertoires of Archaea and the other eukaryotic kingdoms typically ranged 200–260 FSFs and 270–350 FSFs, respectively. This observation is significant. It provides comparative information to support a unique evolutionary link of phyla within superkingdoms Eukarya and Bacteria. Plots of FSF number also clarified functional patterns in outliers, revealing they did not have more numbers of FSFs in *Information* but rather have reduced metabolic repertoires. This shows that parasitic outliers get rid of metabolic domains and become more and more dependent on host cells.

### 2.4. Effect of Organism Lifestyle

The analysis thus far revealed the existence of a small group of outliers within each superkingdom. Manual inspection of lifestyles of these organisms showed that all of these organisms are united by a parasitic or symbiotic lifestyle. For example, *N. equitans* is the smallest archaeal genome ever sequenced and represents a new phylum, the Nanoarchaeaota [28]. This organism interacts with Ignicoccus hospitalis, establishing the only known parasite/symbiont relationship of Archaea, and harbors a highly reduced genome [29]. Parasitic/symbiotic relationships with various plants and animals can be found in Tenericutes and in the endosymbionts of insects that belong to Proteobacteria. Similarly, the *Encephalitozoon* species are eukaryotic parasites that lack mitochondria and have highly reduced genomes [43,44]. *E. cunniculi* has even a chromosomal dispersion of its ribosomal genes, very much like *N. equitans*, and the rRNA of the large ribosomal subunit reduced to its universal core [46]. Similarly, *Guillardia theta* is a nucleomorph that has a highly compact and reduced genome with loss of nearly all metabolic genes [45]. Thus, all outliers exhibit extreme or unique cases of genome reduction.

**Figure 3:**
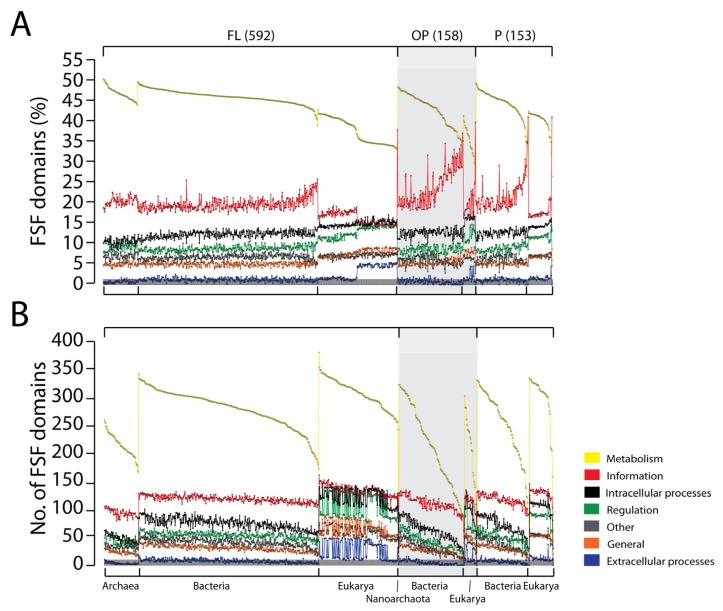
The functional distribution of FSFs with respect to organism lifestyle. Both the percentage (**A**) and actual FSF numbers (**B**) indicate that obligate parasitic (OP) and facultative parasitic (P) organisms exhibit considerable variability in their metabolic repertoires (yellow trend lines) that is offset by corresponding increases in the *Information* FSFs (red trend lines).

In order to explore whether organisms that engage in parasitic or symbiotic interactions have general tendencies that resemble those of the outliers, we classified organisms into three different lifestyles: free living (FL) (592 proteomes), facultative parasitic (P) (153 proteomes), and obligate parasitic (OP) (158 proteomes). Functional distributions for the seven general functional categories for these proteomic sets explained the role of parasitic life on proteomic constitution (Figure 3). Plots of percentages (Figure 3(A)) and actual number of FSFs in proteomes (Figure 3(B)) showed FSF distribution in FL organisms were remarkably homogenous and that the vast majority of variability within superkingdoms was ascribed to the P and OP lifestyles. This variability was for the most part explained by a sharp decline in the number of metabolic FSFs that are assigned to the *Metabolism* general category (Figure 3(B)). Plots also support the hypothesis that parasitic organisms have gone the route of massive genome reduction in a tendency to loose all of their metabolic genes. This tendency makes them more and more dependent on host cells for metabolic functions and survival [47,48].

The number of domains corresponding to each general functional category in the proteomes of FL organisms increases in the order Archaea, Bacteria and Eukarya (Table S3). When compared to the total proteomic set (Figure 2), *Metabolism* remains the predominant functional category and a large number of domains in all the proteomes perform metabolic functions. Again, the proteomes of Eukarya have the richest FSF repertoires, and those of Archaea the most simple. Since maximum variability lies within the proteome repertoires of parasitic/symbiotic organisms (Figure 3) and parasitism/symbiosis in these organisms is the result of secondary adaptations, the analysis of proteomic diversity in FL organisms allows us to test if the functional repertoires of superkingdoms are indeed statistically significant. Analysis of variance showed that the number of FSFs for each functional repertoire was consistently different between superkingdoms (*p* < 0.0001; Table S3). This supports the conclusions drawn from earlier analyses that the microbial superkingdoms followed a genome reduction path while Eukarya expanded their genomic repertoires [7,25].

### 2.5. Analysis of Minor Functional Categories

**Figure 4:**
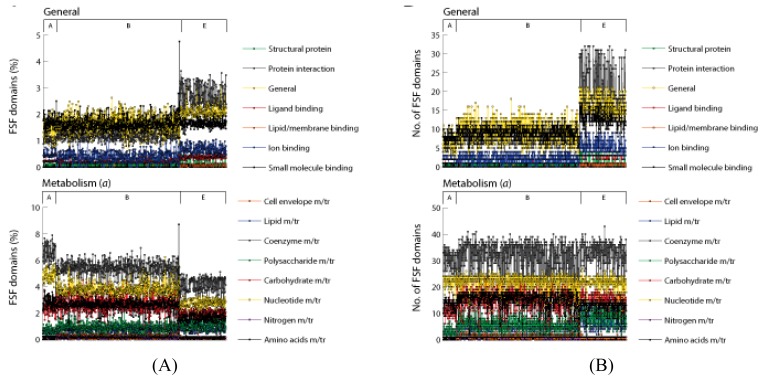

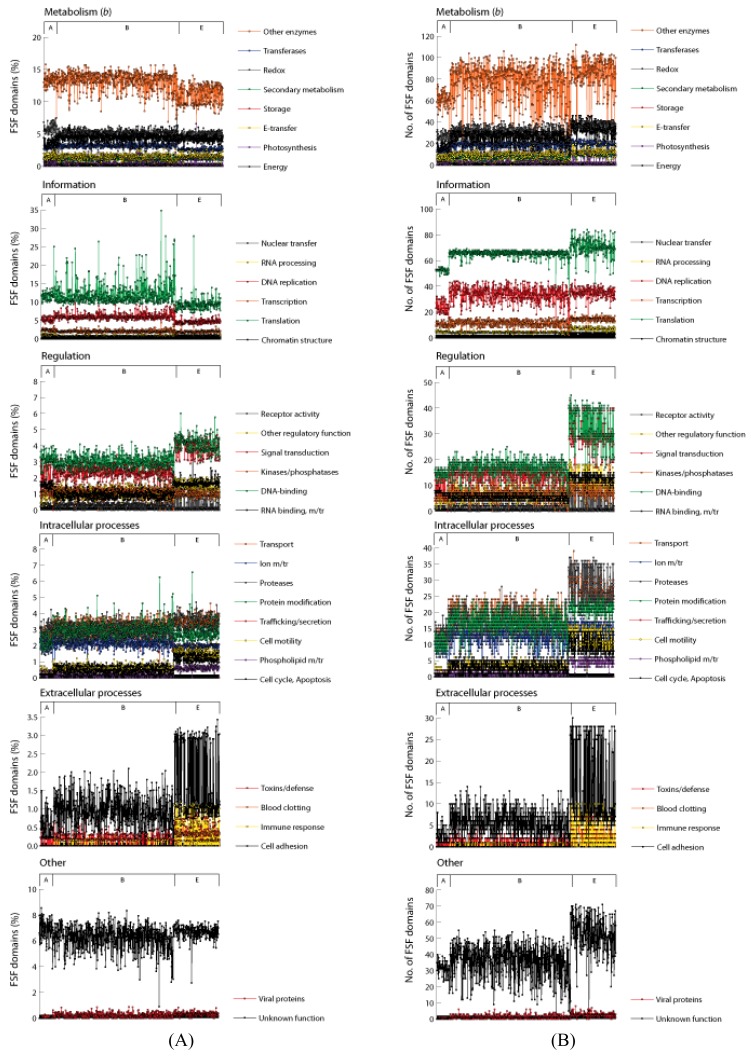
The percentage (**A**) and number (**B**) of FSFs in minor functional categories across superkingdoms. Archaea (**A**) and Bacteria (**B**) spend most of their proteomes in functions related to *Metabolism* and *Information* whereas Eukarya (E) stand out in the minor categories of *Regulation*, *General*, *Intracellular processes* (*ICP)* and *Extracellular processes* *(ECP)*. In turn, the number of FSFs increases in the order Archaea, Bacteria and Eukarya. Eukaryal proteomes have the richest functional repertoires for *Regulation*, *Other*, *General*, *ICP* and *ECP*.

The seven general categories of molecular functions map to 50 minor categories (Table 1). We explored the distribution of FSFs corresponding to each minor category in superkingdoms (Figure 4). Only category “*not annotated*” (*NONA*) was excluded from analysis. In terms of percentage (Figure 4(A)), the overall functional signature is split into two components: prokaryotic and eukaryotic. Prokaryotes spend most of their domain repertoire on *Metabolism* and *Information* whereas Eukarya stand out in *ECP* (particularly cell adhesion, immune response), *Regulation* (DNA binding, signal transduction), and all the minor functional categories corresponding to *ICP* and *General*.

In terms of domain counts (Figure 4(B)), proteomes of Eukarya have the richest functional repertoires with a significantly large number of FSFs devoted for each minor functional category. Bacteria and Archaea work with small number of domains. However, the number of FSFs in Bacteria is significantly higher compared to Archaea (supporting results of Figure 1, Figure 2 and Table S3). These results are consistent with the evolutionary trends in proteomes described previously [7,19,25]. Our results support the complex nature of the Last Universal Common Ancestor (LUCA) [19] and are consistent with the evolution of microbial superkingdoms via reductive evolutionary processes and the evolution of eukaryal proteomes by genome expansion [7,25]. It appears that Archaea went on the route of genome reduction very early in evolution and was followed by Bacteria and finally Eukarya. Late in evolution, the eukaryal superkingdom increased the representation of FSFs and developed a rich proteome. This can explain the relatively huge and diverse nature of eukaryal proteomes compared to prokaryotic proteomes. Finally, there appears to be no significant difference in the distributions of FSFs corresponding to *Metabolism* and *Information* between Bacteria and Eukarya except for minor category “Translation” (green trend lines in Figure 4(B, *Information*)) that is significantly higher in Eukarya compared to Bacteria. This shows that Bacteria exhibit incredible metabolic and informational diversity despite their reduced genomic complements. We conclude that the genome expansion in Eukarya occurred primarily for functions related to *ECP, ICP, Regulation* and *General*.

### 2.6. Reliability of Functional Annotations and Conclusions of this Study

Our analysis depends upon the accuracy of assigning structures to protein sequences and the SCOP protein classification and SUPERFAMILY functional annotation schemes. Databases such as SCOP and SUPERFAMILY are continuously updated with more and more genomes and new assignments. We therefore ask the reader to focus on the general trends in the data as opposed to the specifics such as the exact percentage or numbers of FSFs in each functional repertoire. Trends related to the number of domains in Archaea relative to Bacteria and Eukarya and the reduction of metabolic repertoires in parasitic organisms should be considered robust since these have been reliably observed in previous studies with more limited datasets [1,7,15,19,34]. Biases in sampling of proteomes in the three superkingdoms is not expected to over or underestimate the remarkably conserved nature of the functional makeup. We show that the conservation of molecular functions in proteomes is only broken in genomic outliers that are united by parasitic lifestyles. Thus equal sampling will not significantly alter the global trends described for individual superkingdoms. In light of our results, organism lifestyle is the only factor affecting the conserved nature of proteomes. Finally, we propose that lower or higher than expected numbers of FSFs in any category (subcategory) can be explained either by possible limitations of the scheme used to annotate molecular functions of FSFs or the simple nature of the functional repertoire. For example, the number of FSFs in subcategory structural proteins (main category *General*) is 7 (Table 1) despite the importance of structural proteins in cellular organization. Table S4 lists the description of these FSFs and shows that indeed these FSF domains play important structural roles. Their limited number indicates that the structural and functional organization is quite limited and very few folds play important structural roles. Another possibility is the “hidden” overlap between FSFs and molecular functions due to the one-to-one mapping limitations of the SUPERFAMILY functional annotation scheme. Most of the large FSFs include many FFs and participate in multiple pathways; for few FSFs a complete functional profile may not be intuitively obvious. This may be one of the shortcomings of using this functional annotation scheme but dissection of such detailed functions and pathways is a difficult task and is not described in this study. In summary, we do not believe that the classification or annotation schemes, despite their limitations, would undergo serious revisions or weaken our findings.

## 3. Experimental Section

### 3.1. Data Retrieval

We downloaded the protein architecture assignments for a total of 965 organisms including 70 Archaea, 651 Bacteria and 244 Eukarya (Table S5) from SUPERFAMILY ver. 1.73 MySQL [16,17] at an *E*-value cutoff of 10^−4^. This cutoff is considered a stringent threshold to eliminate the rate of false positives in HMM assignments [19]. Classification of organisms according to their lifestyles was done manually and resulted in 592 FL, 153 P, and 158 OP organisms.

### 3.2. Assigning Functional Categories to Protein Domains

The most recent domain functional annotation file for SCOP 1.73 was downloaded from the SUPERFAMILY webserver [23]. For each genome we extracted the set of unique FSFs present and then mapped them to the 7 general and 50 detailed functional categories. We calculated both the percentage and actual number of domains using programming implementations in Python 3.1 (http://www.python.org/download/).

### 3.3. Statistical Analysis

The statistical significance between the numbers of functional FSFs in FL organisms of superkingdoms was evaluated by Welch's ANOVA in SAS (http://www.sas.com/software/sas9), which is the appropriate test to detect differences between means for groups having unequal variances [49]. We excluded organisms with P and OP lifestyles in order to remove noise from the data. Additionally, in order to meet asymptotic normality, we used the Log10 transformation and rescaled the data to 0–7 using the following formula,

$$N_{\mathrm{normal}}=[{\text{Log}}_{10}(N_{xy})/{\text{Log}}_{10}(N_{\max})]\times 7$$

where *N*_xy_ is the count of a FSF in *x* functional category in *y* superkingdom; *N_max_* is the largest value in the matrix and *N_normal_* is the normalized and scaled score for FSF *x* in *y* superkingdom.

## 4. Conclusions

**Figure S1:**
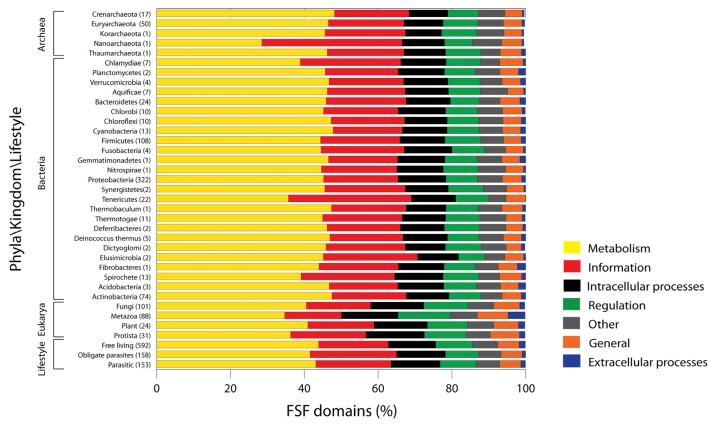
Average distribution of FSFs in phyla, kingdom, and superkingdoms suggest conservation of functional design in proteomes. Numbers in parentheses indicate total number of proteomes analyzed for each phyla/kingdom.

Our analysis revealed a remarkable conservation in the functional distribution of protein domains in superkingdoms for proteomes for which we have structural assignments. Figure S1 showcases average distribution of FSFs in phyla, kingdoms, and superkingdoms. The biggest proportion of each proteome is devoted in all cases to functions related to *Metabolism*. Phylogenomic analysis has shown that *Metabolism* appeared earlier than other functional groups and their structures were the first to spread in life [1,50]. This would explain the relative large representation of *Metabolism* in the functional toolkit of cells. Usage of domains related to *ECP* and *Regulation* is significantly higher in *Metazoa* compared to the rest. This showcases the importance of regulation signal transduction mechanisms for eukaryotic organisms [51,52]. Our results support the view that prokaryotes evolved via reductive evolutionary processes whereas genome expansion was the route taken by eukaryotic organisms. Genome expansion in Eukarya seems to be directed towards innovation of FSF architectures, especially those linked to *Regulation, ECP* and *General*. Finally, viral structures make up a substantial proportion of cellular proteomes and appear to have played an important role in the evolution of cellular life.

Organisms with parasitic lifestyles have simple and reduced proteomes and rely on host cells for metabolic functions. Tenericutes are unique in this regard. They spend most of their proteomic resources in functions linked to *Information* (e.g., translation, replication). Remarkably, we find that the conservation of molecular functions in proteomes is only broken in “outliers” with parasitic lifestyles that do not obey the global trends. We conclude that organism lifestyle is a crucial factor in shaping the nature of proteomes.

## Figures and Tables

**Table 1:** Mapping between the general and minor functional categories for 1,781 protein domains defined in structural classification of proteins (SCOP) 1.73 and the number of fold superfamilies (FSFs) corresponding to each minor category in our dataset of 965 organisms. A total of 135 FSFs could not be annotated. m/tr, metabolism and transport.

**Functional category**	**Minor categories**	**No. of FSF domains**
*Metabolism* (533 FSFs)	Energy	54
	Photosynthesis	20
	E- transfer	31
	Amino acids m/tr	20
	Nitrogen m/tr	1
	Nucleotide m/tr	30
	Carbohydrate m/tr	30
	Polysaccharide m/tr	21
	Storage	0
	Coenzyme m/tr	50
	Lipid m/tr	17
	Cell envelope m/tr	8
	Secondary metabolism	11
	Redox	55
	Transferases	29
	Other enzymes	156
*General* (131 FSFs)	Small molecule binding	27
	Ion binding	13
	Lipid/membrane binding	4
	Ligand binding	3
	General	28
	Protein interaction	49
	Structural protein	7
*Information* (201 FSFs)	Chromatin structure	7
	Translation	92
	Transcription	24
	DNA replication/repair	68
	RNA processing	10
	Nuclear structure	0
*Other* (273 FSFs)	Unknown function	200
	Viral proteins	73
*Extracellular processes* (95 FSFs)	Cell adhesion	31
	Immune response	19
	Blood clotting	5
	Toxins/defense	40
*Intracellular processes* (208 FSFs)	Cell cycle, Apoptosis	20
	Phospholipid m/tr	6
	Cell motility	20
	Trafficking/secretion	0
	Protein modification	35
	Proteases	52
	Ion m/tr	21
	Transport	54
*Regulation* (205 FSFs)	RNA binding, m/tr	19
	DNA-binding	66
	Kinases/phosphatases	15
	Signal transduction	53
	Other regulatory function	34
	Receptor activity	18

## Supplementary Materials

**Table S1:** Average number of FSF domains in each phyla/kingdom corresponding to the seven general functional categories. Numbers were rounded up when the decimal value exceeded 0.5 and rounded down otherwise. Nanoarchaeota and Tenericutes have the least number of metabolic domains and are highlighted in bold. Eukaryal kingdoms (Fungi, Metazoa, Plants and Protista) have the richest FSF repertoires compared to the prokaryotes.

**Superkingdom**	**Phyla/Kingdom**	**Metabolism**	**Information**	**ICP**	**Regulation**	**Other**	**General**	**ECP**
**Archaea**	Crenarchaeota	204	85	44	35	30	20	2
	Euryarchaeota	219	96	50	44	32	24	4
	Korarchaeota	178	85	38	37	29	19	2
	**Nanoarchaeota**	**57**	**76**	**23**	**15**	**16**	**11**	**1**
	Thaumarchaeota	202	91	49	42	23	25	5
**Bacteria**	Proteobacteria	274	119	78	52	42	31	7
	Firmicutes	246	117	67	53	35	26	7
	Actinobacteria	275	115	66	50	33	30	7
	Bacteroidetes	251	113	65	43	32	29	9
	**Tenericutes**	**99**	**90**	**33**	**25**	**13**	**14**	**0**
	Cyanobacteria	289	112	73	52	39	30	8
	Spirochaetes	171	104	56	41	24	25	5
	Thermotogae	231	110	60	48	36	22	4
	Rest of Bacteria *	255	113	67	48	37	27	6
	PVC	206	110	58	43	28	27	6
**Eukarya**	Fungi	298	127	105	87	51	52	10
	Metazoa	307	135	136	126	65	75	42
	Plants	332	145	117	87	58	54	14
	Protista	220	117	94	67	39	46	9

* Includes proteomes from Chlorobi, Chloroflexi, Aquificae, Deinococcus thermus, Fusobacteria, Acidobacteria, Deferribacters, Dictyoglomi, Elusimicrobia, Synergistetes, Fibrobacters, Gemmatimonadetes, Nitrospirae, and Thermobaculum.

**Table S2:** Average percentage of FSF domains in each phyla/kingdom corresponding to the seven general functional categories. Numbers were rounded up when the decimal value exceeded 0.5 and rounded down otherwise. Nanoarchaeota (highlighted in bold) is an outlier considering it has the smallest percentage for metabolic domains compared to the rest and this decrease is offset by an increase in the informational FSFs.

**Superkingdom**	**Phyla/Kingdom**	**Metabolism**	**Information**	**ICP**	**Regulation**	**Other**	**General**	**ECP**
**Archaea**	Crenarchaeota	48	21	10	9	7	5	1
	Euryarchaeota	47	20	11	9	7	5	1
	Korarchaeota	46	22	10	9	7	5	1
	**Nanoarchaeota**	**29**	**38**	**12**	**8**	**8**	**6**	**1**
	Thaumarchaeota	46	21	11	10	5	6	1
**Bacteria**	Proteobacteria	45	20	13	8	7	5	1
	Firmicutes	44	21	12	10	6	5	1
	Actinobacteria	48	20	12	9	6	5	1
	Bacteroidetes	46	22	12	8	6	5	2
	Tenericutes	36	33	12	9	5	5	0
**Bacteria**	Cyanobacteria	48	19	12	9	6	5	1
	Spirochaetes	39	25	13	10	6	6	1
	Thermotogae	45	22	12	9	7	4	1
	Rest of Bacteria *	46	21	12	9	7	5	1
	PVC	42	24	12	9	6	6	1
**Eukarya**	Fungi	41	17	14	12	7	7	1
	Metazoa	35	15	15	14	7	8	5
	Plants	41	18	14	11	7	7	2
	Protista	36	20	16	11	6	8	2

* Includes proteomes from Chlorobi, Chloroflexi, Aquificae, Deinococcus thermus, Fusobacteria, Acidobacteria, Deferribacters, Dictyoglomi, Elusimicrobia, Synergistetes, Fibrobacters, Gemmatimonadetes, Nitrospirae, and Thermobaculum

**Table S3:** Comparison of functional categories across superkingdoms using Welch's ANOVA.

**Functional category**	**F-ratio**	**DF**	*P***-value** *****
*Metabolism*	350.21	2	<0.0001
*Information*	582.28	2	<0.0001
*ICP*	1271.32	2	<0.0001
*Regulation*	966.75	2	<0.0001
*Other*	520.97	2	<0.0001
*General*	1043.76	2	<0.0001
*ECP*	263.44	2	<0.0001

* All the *P*-values are statistically significant at 0.05.

**Table S4:** Names and description of FSF domains corresponding to subcategory *structural proteins* in the main category *General*.

**No.**	**SCOP Id**	**FSF Id**	**Description**
1	103589	g.71.1	Mini-collagen I, C-terminal domain
2	49695	b.11.1	Gamma-crystallin-like
3	51269	b.85.1	Anti-freeze protein (AFP) III-like domain
4	56558	d.182.1	Baseplate structural protein gp11
5	58002	h.1.6	Chicken cartilage matrix protein
6	58006	h.1.7	Assembly domain of catrillage oligomeric matrix protein
7	75404	d.213.1	Vesiculovirus (VSV) matrix proteins

**Table S5:** List of organisms analyzed with their taxonomic classifications.

**No.**	***Genome Name***	**Phyla/Kingdom**	**Superkingdom**
1	*Malassezia globosa CBS 7966*	Fungi	Eukaryota
2	*Ustilago maydis*	Fungi	Eukaryota
3	*Puccinia graminis f. sp. tritici CRL 75-36-700-3*	Fungi	Eukaryota
4	*Melampsora laricis-populina*	Fungi	Eukaryota
5	*Sporobolomyces roseus IAM 13481*	Fungi	Eukaryota
6	*Serpula lacrymans var. lacrymans S7.9*	Fungi	Eukaryota
7	*Coprinopsis cinerea okayama7 130 v3*	Fungi	Eukaryota
8	*Pleurotus ostreatus*	Fungi	Eukaryota
9	*Laccaria bicolor S238N-H82*	Fungi	Eukaryota
10	*Agaricus bisporus var. bisporus*	Fungi	Eukaryota
11	*Schizophyllum commune*	Fungi	Eukaryota
12	*Heterobasidion annosum*	Fungi	Eukaryota
13	*Phanerochaete chrysosporium RP-78 2.1*	Fungi	Eukaryota
14	*Postia placenta*	Fungi	Eukaryota
15	*Tremella mesenterica*	Fungi	Eukaryota
16	*Cryptococcus neoformans JEC21*	Fungi	Eukaryota
17	*Magnaporthe grisea 70-15*	Fungi	Eukaryota
18	*Podospora anserina*	Fungi	Eukaryota
19	*Sporotrichum thermophile ATCC 42464*	Fungi	Eukaryota
20	*Thielavia terrestris NRRL 8126*	Fungi	Eukaryota
21	*Chaetomium globosum CBS 148.51*	Fungi	Eukaryota
22	*Neurospora tetrasperma*	Fungi	Eukaryota
23	*Neurospora discreta FGSC 8579*	Fungi	Eukaryota
24	*Neurospora crassa OR74A*	Fungi	Eukaryota
25	*Cryphonectria parasitica*	Fungi	Eukaryota
26	*Verticillium dahliae VdLs.17*	Fungi	Eukaryota
27	*Verticillium albo-atrum VaMs.102*	Fungi	Eukaryota
28	*Fusarium oxysporum f. sp. lycopersici 4286*	Fungi	Eukaryota
29	*Nectria haematococca mpVI*	Fungi	Eukaryota
30	*Fusarium verticillioides 7600*	Fungi	Eukaryota
31	*Fusarium graminearum*	Fungi	Eukaryota
32	*Trichoderma atroviride*	Fungi	Eukaryota
33	*Trichoderma reesei 1.2*	Fungi	Eukaryota
34	*Trichoderma virens Gv29-8*	Fungi	Eukaryota
35	*Botrytis cinerea B05.10*	Fungi	Eukaryota
36	*Sclerotinia sclerotiorum*	Fungi	Eukaryota
37	*Alternaria brassicicola*	Fungi	Eukaryota
38	*Pyrenophora tritici-repentis*	Fungi	Eukaryota
39	*Cochliobolus heterostrophus*	Fungi	Eukaryota
40	*Stagonospora nodorum*	Fungi	Eukaryota
41	*Mycosphaerella fijiensis CIRAD86*	Fungi	Eukaryota
42	*Mycosphaerella graminicola IPO323*	Fungi	Eukaryota
43	*Ajellomyces dermatitidis SLH14081*	Fungi	Eukaryota
44	*Histoplasma capsulatum class NAmI strain WU24*	Fungi	Eukaryota
45	*Microsporum canis CBS 113480*	Fungi	Eukaryota
46	*Microsporum gypseum*	Fungi	Eukaryota
47	*Arthroderma benhamiae CBS 112371*	Fungi	Eukaryota
48	*Trichophyton equinum CBS 127.97*	Fungi	Eukaryota
49	*Trichophyton verrucosum HKI 0517*	Fungi	Eukaryota
50	*Trichophyton tonsurans CBS 112818*	Fungi	Eukaryota
51	*Trichophyton rubrum CBS 118892*	Fungi	Eukaryota
52	*Paracoccidioides brasiliensis Pb18*	Fungi	Eukaryota
53	*Coccidioides posadasii RMSCC 3488*	Fungi	Eukaryota
54	*Coccidioides immitis RS*	Fungi	Eukaryota
55	*Uncinocarpus reesii 1704*	Fungi	Eukaryota
56	*Aspergillus fumigatus Af293*	Fungi	Eukaryota
57	*Neosartorya fischeri NRRL 181*	Fungi	Eukaryota
58	*Penicillium chrysogenum Wisconsin 54-1255*	Fungi	Eukaryota
59	*Penicillium marneffei ATCC 18224*	Fungi	Eukaryota
60	*Aspergillus carbonarius ITEM 5010*	Fungi	Eukaryota
61	*Aspergillus terreus NIH2624*	Fungi	Eukaryota
62	*Aspergillus oryzae RIB40*	Fungi	Eukaryota
63	*Aspergillus niger ATCC 1015*	Fungi	Eukaryota
64	*Aspergillus flavus NRRL3357*	Fungi	Eukaryota
65	*Aspergillus clavatus NRRL 1*	Fungi	Eukaryota
66	*Aspergillus nidulans FGSC A4*	Fungi	Eukaryota
67	*Tuber melanosporum Vittad*	Fungi	Eukaryota
68	*Pichia stipitis CBS 6054*	Fungi	Eukaryota
69	*Candida guilliermondii ATCC 6260*	Fungi	Eukaryota
70	*Lodderomyces elongisporus NRRL YB-4239*	Fungi	Eukaryota
71	*Debaromyces hansenii*	Fungi	Eukaryota
72	*Candida dubliniensis CD36*	Fungi	Eukaryota
73	*Candida tropicalis MYA-3404*	Fungi	Eukaryota
74	*Candida parapsilosis*	Fungi	Eukaryota
75	*Candida albicans SC5314*	Fungi	Eukaryota
76	*Yarrowia lipolytica CLIB122*	Fungi	Eukaryota
77	*Candida lusitaniae ATCC 42720*	Fungi	Eukaryota
78	*Vanderwaltozyma polyspora DSM 70294*	Fungi	Eukaryota
79	*Candida glabrata CBS138*	Fungi	Eukaryota
80	*Kluyveromyces thermotolerans CBS 6340*	Fungi	Eukaryota
81	*Lachancea kluyveri*	Fungi	Eukaryota
82	*Kluyveromyces waltii*	Fungi	Eukaryota
83	*Ashbya gossypii ATCC 10895*	Fungi	Eukaryota
84	*Zygosaccharomyces rouxii*	Fungi	Eukaryota
85	*Saccharomyces mikatae MIT*	Fungi	Eukaryota
86	*Saccharomyces paradoxus MIT*	Fungi	Eukaryota
87	*Saccharomyces cerevisiae SGD*	Fungi	Eukaryota
88	*Saccharomyces bayanus MIT*	Fungi	Eukaryota
89	*Pichia pastoris GS115*	Fungi	Eukaryota
90	*Kluyveromyces lactis*	Fungi	Eukaryota
91	*Schizosaccharomyces octosporus yFS286*	Fungi	Eukaryota
92	*Schizosaccharomyces japonicus yFS275*	Fungi	Eukaryota
93	*Schizosaccharomyces pombe*	Fungi	Eukaryota
94	*Allomyces macrogynus ATCC 38327*	Fungi	Eukaryota
95	*Rhizopus oryzae RA 99-880*	Fungi	Eukaryota
96	*Phycomyces blakesleeanus*	Fungi	Eukaryota
97	*Mucor circinelloides*	Fungi	Eukaryota
98	*Spizellomyces punctatus DAOM BR117*	Fungi	Eukaryota
99	*Batrachochytrium dendrobatidis JEL423*	Fungi	Eukaryota
100	*Encephalitozoon cuniculi*	Fungi	Eukaryota
101	*Encephalitozoon intestinalis*	Fungi	Eukaryota
102	*Homo sapiens 59_37d (all transcripts)*	Metazoa	Eukaryota
103	*Pan troglodytes 59_21n (all transcripts)*	Metazoa	Eukaryota
104	*Gorilla gorilla 59_3b (all transcripts)*	Metazoa	Eukaryota
105	*Pongo pygmaeus 59_1e (all transcripts)*	Metazoa	Eukaryota
106	*Macaca mulatta 59_10n (all transcripts)*	Metazoa	Eukaryota
107	*Callithrix jacchus 59_321a (all transcripts)*	Metazoa	Eukaryota
108	*Otolemur garnettii 59_1g (all transcripts)*	Metazoa	Eukaryota
109	*Microcebus murinus 59_1d (all transcripts)*	Metazoa	Eukaryota
110	*Tarsius syrichta 59_1e (all transcripts)*	Metazoa	Eukaryota
111	*Rattus norvegicus 59_34a (all transcripts)*	Metazoa	Eukaryota
112	*Mus musculus 59_37l (all transcripts)*	Metazoa	Eukaryota
113	*Spermophilus tridecemlineatus 59_1i (all transcripts)*	Metazoa	Eukaryota
114	*Dipodomys ordii 59_1e (all transcripts)*	Metazoa	Eukaryota
115	*Cavia porcellus 59_3c (all transcripts)*	Metazoa	Eukaryota
116	*Oryctolagus cuniculus 59_2b (all transcripts)*	Metazoa	Eukaryota
117	*Ochotona princeps 59_1e (all transcripts)*	Metazoa	Eukaryota
118	*Tupaia belangeri 59_1h (all transcripts)*	Metazoa	Eukaryota
119	*Sus scrofa 59_9c (all transcripts)*	Metazoa	Eukaryota
120	*Bos taurus 59_4h (all transcripts)*	Metazoa	Eukaryota
121	*Vicugna pacos 59_1e (all transcripts)*	Metazoa	Eukaryota
122	*Tursiops truncatus 59_1e (all transcripts)*	Metazoa	Eukaryota
123	*Canis familiaris 59_2o (all transcripts)*	Metazoa	Eukaryota
124	*Felis catus 59_1h (all transcripts)*	Metazoa	Eukaryota
125	*Equus caballus 59_2f (all transcripts)*	Metazoa	Eukaryota
126	*Myotis lucifugus 59_1i (all transcripts)*	Metazoa	Eukaryota
127	*Pteropus vampyrus 59_1e (all transcripts)*	Metazoa	Eukaryota
128	*Sorex araneus 59_1g (all transcripts)*	Metazoa	Eukaryota
129	*Erinaceus europaeus 59_1g (all transcripts)*	Metazoa	Eukaryota
130	*Procavia capensis 59_1e (all transcripts)*	Metazoa	Eukaryota
131	*Loxodonta africana 59_3b (all transcripts)*	Metazoa	Eukaryota
132	*Echinops telfairi 59_1i (all transcripts)*	Metazoa	Eukaryota
133	*Dasypus novemcinctus 59_2c (all transcripts)*	Metazoa	Eukaryota
134	*Macropus eugenii 59_1b (all transcripts)*	Metazoa	Eukaryota
135	*Monodelphis domestica 59_5k (all transcripts)*	Metazoa	Eukaryota
136	*Ornithorhynchus anatinus 59_1m (all transcripts)*	Metazoa	Eukaryota
137	*Anolis carolinensis 59_1c (all transcripts)*	Metazoa	Eukaryota
138	*Taeniopygia guttata 59_1e (all transcripts)*	Metazoa	Eukaryota
139	*Meleagris gallopavo 57_2 (all transcripts)*	Metazoa	Eukaryota
140	*Gallus gallus 59_2o (all transcripts)*	Metazoa	Eukaryota
141	*Xenopus laevis*	Metazoa	Eukaryota
142	*Xenopus tropicalis 59_41p (all transcripts)*	Metazoa	Eukaryota
143	*Danio rerio 59_8e (all transcripts)*	Metazoa	Eukaryota
144	*Gasterosteus aculeatus 59_1l (all transcripts)*	Metazoa	Eukaryota
145	*Oryzias latipes 59_1k (all transcripts)*	Metazoa	Eukaryota
146	*Tetraodon nigroviridis 59_8d (all transcripts)*	Metazoa	Eukaryota
147	*Takifugu rubripes 59_4m (all transcripts)*	Metazoa	Eukaryota
148	*Branchiostoma floridae 1.0*	Metazoa	Eukaryota
149	*Ciona savignyi 59_2j (all transcripts)*	Metazoa	Eukaryota
150	*Ciona intestinalis 59_2o (all transcripts)*	Metazoa	Eukaryota
151	*Strongylocentrotus purpuratus*	Metazoa	Eukaryota
152	*Helobdella robusta*	Metazoa	Eukaryota
153	*Capitella sp. I*	Metazoa	Eukaryota
154	*Bombyx mori*	Metazoa	Eukaryota
155	*Nasonia vitripennis*	Metazoa	Eukaryota
156	*Apis mellifera 38.2d (all transcripts)*	Metazoa	Eukaryota
157	*Drosophila grimshawi 1.3*	Metazoa	Eukaryota
158	*Drosophila willistoni 1.3*	Metazoa	Eukaryota
159	*Drosophila pseudoobscura 2.13*	Metazoa	Eukaryota
160	*Drosophila persimilis 1.3*	Metazoa	Eukaryota
161	*Drosophila yakuba 1.3*	Metazoa	Eukaryota
162	*Drosophila simulans 1.3*	Metazoa	Eukaryota
163	*Drosophila sechellia 1.3*	Metazoa	Eukaryota
164	*Drosophila melanogaster 59_525a (all transcripts)*	Metazoa	Eukaryota
165	*Drosophila erecta 1.3*	Metazoa	Eukaryota
166	*Drosophila ananassae 1.3*	Metazoa	Eukaryota
167	*Drosophila virilis 1.2*	Metazoa	Eukaryota
168	*Drosophila mojavensis 1.3*	Metazoa	Eukaryota
169	*Aedes aegypti 55 (all transcripts)*	Metazoa	Eukaryota
170	*Culex pipiens quinquefasciatus*	Metazoa	Eukaryota
171	*Anopheles gambiae 49_3j (all transcripts)*	Metazoa	Eukaryota
172	*Tribolium castaneum 3.0*	Metazoa	Eukaryota
173	*Pediculus humanus corporis*	Metazoa	Eukaryota
174	*Acyrthosiphon pisum*	Metazoa	Eukaryota
175	*Daphnia pulex*	Metazoa	Eukaryota
176	*Ixodes scapularis*	Metazoa	Eukaryota
177	*Lottia gigantea*	Metazoa	Eukaryota
178	*Pristionchus pacificus*	Metazoa	Eukaryota
179	*Meloidogyne incognita*	Metazoa	Eukaryota
180	*Brugia malayi WS218*	Metazoa	Eukaryota
181	*Caenorhabditis japonica*	Metazoa	Eukaryota
182	*Caenorhabditis brenneri*	Metazoa	Eukaryota
183	*Caenorhabditis remanei*	Metazoa	Eukaryota
184	*Caenorhabditis elegans 59_210a (all transcripts)*	Metazoa	Eukaryota
185	*Caenorhabditis briggsae 2*	Metazoa	Eukaryota
186	*Schistosoma mansoni*	Metazoa	Eukaryota
187	*Nematostella vectensis 1.0*	Metazoa	Eukaryota
188	*Hydra magnipapillata*	Metazoa	Eukaryota
189	*Trichoplax adhaerens*	Metazoa	Eukaryota
190	*Giardia lamblia 2.3*	Protista	Eukaryota
191	*Trypanosoma cruzi strain CL Brener*	Protista	Eukaryota
192	*Trypanosoma brucei*	Protista	Eukaryota
193	*Leishmania mexicana 2.4*	Protista	Eukaryota
194	*Leishmania major strain Friedlin*	Protista	Eukaryota
195	*Leishmania infantum JPCM5 2.4*	Protista	Eukaryota
196	*Leishmania braziliensis MHOM/BR/75/M2904 2.4*	Protista	Eukaryota
197	*Aureococcus anophagefferens*	Protista	Eukaryota
198	*Phytophthora ramorum 1.1*	Protista	Eukaryota
199	*Phytophthora sojae 1.1*	Protista	Eukaryota
200	*Phytophthora infestans T30-4*	Protista	Eukaryota
201	*Phytophthora capsici*	Protista	Eukaryota
202	*Paramecium tetraurelia*	Protista	Eukaryota
203	*Tetrahymena thermophila SB210 1*	Protista	Eukaryota
204	*Babesia bovis T2Bo*	Protista	Eukaryota
205	*Theileria parva*	Protista	Eukaryota
206	*Theileria annulata*	Protista	Eukaryota
207	*Plasmodium falciparum 3D7*	Protista	Eukaryota
208	*Plasmodium vivax SaI-1 7.0*	Protista	Eukaryota
209	*Plasmodium knowlesi strain H*	Protista	Eukaryota
210	*Plasmodium yoelii ssp. yoelii 1*	Protista	Eukaryota
211	*Plasmodium chabaudi*	Protista	Eukaryota
212	*Plasmodium berghei ANKA*	Protista	Eukaryota
213	*Cryptosporidium hominis*	Protista	Eukaryota
214	*Cryptosporidium muris*	Protista	Eukaryota
215	*Cryptosporidium parvum Iowa II*	Protista	Eukaryota
216	*Neospora caninum Nc-Liverpool 6.2*	Protista	Eukaryota
217	*Neospora caninum*	Protista	Eukaryota
218	*Toxoplasma gondii ME49*	Protista	Eukaryota
219	*Naegleria gruberi*	Protista	Eukaryota
220	*Guillardia theta*	Protista	Eukaryota
221	*Arabidopsis lyrata*	Plantae	Eukaryota
222	*Arabidopsis thaliana 10 (all transcripts)*	Plantae	Eukaryota
223	*Carica papaya*	Plantae	Eukaryota
224	*Medicago truncatula*	Plantae	Eukaryota
225	*Glycine max*	Plantae	Eukaryota
226	*Cucumis sativus*	Plantae	Eukaryota
227	*Populus trichocarpa 6.0*	Plantae	Eukaryota
228	*Vitis vinifera*	Plantae	Eukaryota
229	*Brachypodium distachyon*	Plantae	Eukaryota
230	*Oryza sativa ssp. japonica 5.0*	Plantae	Eukaryota
231	*Zea mays subsp. mays*	Plantae	Eukaryota
232	*Sorghum bicolor*	Plantae	Eukaryota
233	*Selaginella moellendorffii*	Plantae	Eukaryota
234	*Physcomitrella patens subsp. patens*	Plantae	Eukaryota
235	*Ostreococcus sp. RCC809*	Plantae	Eukaryota
236	*Ostreococcus lucimarinus CCE9901*	Plantae	Eukaryota
237	*Ostreococcus tauri*	Plantae	Eukaryota
238	*Micromonas sp. RCC299*	Plantae	Eukaryota
239	*Micromonas pusilla CCMP1545*	Plantae	Eukaryota
240	*Coccomyxa sp. C-169*	Plantae	Eukaryota
241	*Chlorella sp. NC64A*	Plantae	Eukaryota
242	*Chlorella vulgaris*	Plantae	Eukaryota
243	*Volvox carteri f. nagariensis*	Plantae	Eukaryota
244	*Chlamydomonas reinhardtii 4.0*	Plantae	Eukaryota
245	*Candidatus Koribacter versatilis Ellin345*	Acidobacteria	Bacteria
246	*Candidatus Solibacter usitatus Ellin6076*	Acidobacteria	Bacteria
247	*Acidobacterium capsulatum ATCC 51196*	Acidobacteria	Bacteria
248	*Gardnerella vaginalis 409-05*	Actinobacteria	Bacteria
249	*Bifidobacterium longum NCC2705*	Actinobacteria	Bacteria
250	*Bifidobacterium animalis ssp. lactis AD011*	Actinobacteria	Bacteria
251	*Bifidobacterium dentium Bd1*	Actinobacteria	Bacteria
252	*Bifidobacterium adolescentis ATCC 15703*	Actinobacteria	Bacteria
253	*Kineococcus radiotolerans SRS30216*	Actinobacteria	Bacteria
254	*Catenulispora acidiphila DSM 44928*	Actinobacteria	Bacteria
255	*Stackebrandtia nassauensis DSM 44728*	Actinobacteria	Bacteria
256	*Acidothermus cellulolyticus 11B*	Actinobacteria	Bacteria
257	*Nakamurella multipartita DSM 44233*	Actinobacteria	Bacteria
258	*Geodermatophilus obscurus DSM 43160*	Actinobacteria	Bacteria
259	*Frankia sp. CcI3*	Actinobacteria	Bacteria
260	*Frankia alni ACN14a*	Actinobacteria	Bacteria
261	*Thermobifida fusca YX*	Actinobacteria	Bacteria
262	*Thermomonospora curvata DSM 43183*	Actinobacteria	Bacteria
263	*Streptosporangium roseum DSM 43021*	Actinobacteria	Bacteria
264	*Streptomyces griseus ssp. griseus NBRC 13350*	Actinobacteria	Bacteria
265	*Streptomyces avermitilis MA-4680*	Actinobacteria	Bacteria
266	*Streptomyces scabiei 87.22*	Actinobacteria	Bacteria
267	*Streptomyces coelicolor*	Actinobacteria	Bacteria
268	*Actinosynnema mirum DSM 43827*	Actinobacteria	Bacteria
269	*Saccharomonospora viridis DSM 43017*	Actinobacteria	Bacteria
270	*Saccharopolyspora erythraea NRRL 2338*	Actinobacteria	Bacteria
271	*Kribbella flavida DSM 17836*	Actinobacteria	Bacteria
272	*Nocardioides sp. JS614*	Actinobacteria	Bacteria
273	*Propionibacterium acnes KPA171202*	Actinobacteria	Bacteria
274	*Salinispora arenicola CNS-205*	Actinobacteria	Bacteria
275	*Salinispora tropica CNB-440*	Actinobacteria	Bacteria
276	*Gordonia bronchialis DSM 43247*	Actinobacteria	Bacteria
277	*Rhodococcus jostii RHA1*	Actinobacteria	Bacteria
278	*Rhodococcus opacus B4*	Actinobacteria	Bacteria
279	*Rhodococcus erythropolis PR4*	Actinobacteria	Bacteria
280	*Nocardia farcinica IFM 10152*	Actinobacteria	Bacteria
281	*Mycobacterium abscessus ATCC 19977*	Actinobacteria	Bacteria
282	*Mycobacterium sp. MCS*	Actinobacteria	Bacteria
283	*Mycobacterium avium ssp. paratuberculosis K-10*	Actinobacteria	Bacteria
284	*Mycobacterium vanbaalenii PYR-1*	Actinobacteria	Bacteria
285	*Mycobacterium tuberculosis H37Rv*	Actinobacteria	Bacteria
286	*Mycobacterium bovis AF2122/97*	Actinobacteria	Bacteria
287	*Mycobacterium ulcerans Agy99*	Actinobacteria	Bacteria
288	*Mycobacterium gilvum PYR-GCK*	Actinobacteria	Bacteria
289	*Mycobacterium marinum M*	Actinobacteria	Bacteria
290	*Mycobacterium smegmatis MC2 155*	Actinobacteria	Bacteria
291	*Mycobacterium leprae TN*	Actinobacteria	Bacteria
292	*Corynebacterium aurimucosum ATCC 700975*	Actinobacteria	Bacteria
293	*Corynebacterium kroppenstedtii DSM 44385*	Actinobacteria	Bacteria
294	*Corynebacterium efficiens YS-314*	Actinobacteria	Bacteria
295	*Corynebacterium urealyticum DSM 7109*	Actinobacteria	Bacteria
296	*Corynebacterium jeikeium K411*	Actinobacteria	Bacteria
297	*Corynebacterium glutamicum ATCC 13032 Kitasato*	Actinobacteria	Bacteria
298	*Corynebacterium diphtheriae NCTC 13129*	Actinobacteria	Bacteria
299	*Tropheryma whipplei Twist*	Actinobacteria	Bacteria
300	*Sanguibacter keddieii DSM 10542*	Actinobacteria	Bacteria
301	*Kytococcus sedentarius DSM 20547*	Actinobacteria	Bacteria
302	*Beutenbergia cavernae DSM 12333*	Actinobacteria	Bacteria
303	*Leifsonia xyli ssp. xyli CTCB07*	Actinobacteria	Bacteria
304	*Clavibacter michiganensis ssp. michiganensis NCPPB 382*	Actinobacteria	Bacteria
305	*Jonesia denitrificans DSM 20603*	Actinobacteria	Bacteria
306	*Brachybacterium faecium DSM 4810*	Actinobacteria	Bacteria
307	*Xylanimonas cellulosilytica DSM 15894*	Actinobacteria	Bacteria
308	*Kocuria rhizophila DC2201*	Actinobacteria	Bacteria
309	*Rothia mucilaginosa DY-18*	Actinobacteria	Bacteria
310	*Arthrobacter sp. FB24*	Actinobacteria	Bacteria
311	*Arthrobacter chlorophenolicus A6*	Actinobacteria	Bacteria
312	*Arthrobacter aurescens TC1*	Actinobacteria	Bacteria
313	*Renibacterium salmoninarum ATCC 33209*	Actinobacteria	Bacteria
314	*Micrococcus luteus NCTC 2665*	Actinobacteria	Bacteria
315	*Cryptobacterium curtum DSM 15641*	Actinobacteria	Bacteria
316	*Eggerthella lenta DSM 2243*	Actinobacteria	Bacteria
317	*Slackia heliotrinireducens DSM 20476*	Actinobacteria	Bacteria
318	*Atopobium parvulum DSM 20469*	Actinobacteria	Bacteria
319	*Conexibacter woesei DSM 14684*	Actinobacteria	Bacteria
320	*Rubrobacter xylanophilus DSM 9941*	Actinobacteria	Bacteria
321	*Acidimicrobium ferrooxidans DSM 10331*	Actinobacteria	Bacteria
322	*Sulfurihydrogenibium sp. YO3AOP1*	Aquificae	Bacteria
323	*Sulfurihydrogenibium azorense Az-Fu1*	Aquificae	Bacteria
324	*Persephonella marina EX-H1*	Aquificae	Bacteria
325	*Hydrogenobaculum sp. Y04AAS1*	Aquificae	Bacteria
326	*Thermocrinis albus DSM 14484*	Aquificae	Bacteria
327	*Aquifex aeolicus VF5*	Aquificae	Bacteria
328	*Hydrogenobacter thermophilus TK-6*	Aquificae	Bacteria
329	*Dyadobacter fermentans DSM 18053*	Bacteroidetes	Bacteria
330	*Cytophaga hutchinsonii ATCC 33406*	Bacteroidetes	Bacteria
331	*Spirosoma linguale DSM 74*	Bacteroidetes	Bacteria
332	*Candidatus Azobacteroides pseudotrichonymphae genomovar.*	Bacteroidetes	Bacteria
333	*Prevotella ruminicola 23*	Bacteroidetes	Bacteria
334	*Parabacteroides distasonis ATCC 8503*	Bacteroidetes	Bacteria
335	*Porphyromonas gingivalis W83*	Bacteroidetes	Bacteria
336	*Bacteroides vulgatus ATCC 8482*	Bacteroidetes	Bacteria
337	*Bacteroides thetaiotaomicron VPI-5482*	Bacteroidetes	Bacteria
338	*Bacteroides fragilis NCTC 9343*	Bacteroidetes	Bacteria
339	*Candidatus Amoebophilus asiaticus 5a2*	Bacteroidetes	Bacteria
340	*Salinibacter ruber DSM 13855*	Bacteroidetes	Bacteria
341	*Rhodothermus marinus DSM 4252*	Bacteroidetes	Bacteria
342	*Chitinophaga pinensis DSM 2588*	Bacteroidetes	Bacteria
343	*Pedobacter heparinus DSM 2366*	Bacteroidetes	Bacteria
344	*Candidatus Sulcia muelleri GWSS*	Bacteroidetes	Bacteria
345	*Zunongwangia profunda SM-A87*	Bacteroidetes	Bacteria
346	*Gramella forsetii KT0803*	Bacteroidetes	Bacteria
347	*Robiginitalea biformata HTCC2501*	Bacteroidetes	Bacteria
348	*Flavobacteriaceae bacterium 3519-10*	Bacteroidetes	Bacteria
349	*Capnocytophaga ochracea DSM 7271*	Bacteroidetes	Bacteria
350	*Flavobacterium psychrophilum JIP02/86*	Bacteroidetes	Bacteria
351	*Flavobacterium johnsoniae UW101*	Bacteroidetes	Bacteria
352	*Blattabacterium sp. Bge*	Bacteroidetes	Bacteria
353	*Candidatus Protochlamydia amoebophila UWE25*	Chlamydiae	Bacteria
354	*Chlamydophila pneumoniae TW-183*	Chlamydiae	Bacteria
355	*Chlamydophila caviae GPIC*	Chlamydiae	Bacteria
356	*Chlamydophila felis Fe/C-56*	Chlamydiae	Bacteria
357	*Chlamydophila abortus S26/3*	Chlamydiae	Bacteria
358	*Chlamydia muridarum Nigg*	Chlamydiae	Bacteria
359	*Chlamydia trachomatis D/UW-3/CX*	Chlamydiae	Bacteria
360	*Pelodictyon phaeoclathratiforme BU-1*	Chlorobi	Bacteria
361	*Chlorobium luteolum DSM 273*	Chlorobi	Bacteria
362	*Chlorobium chlorochromatii CaD3*	Chlorobi	Bacteria
363	*Chlorobium phaeobacteroides DSM 266*	Chlorobi	Bacteria
364	*Chlorobium phaeovibrioides DSM 265*	Chlorobi	Bacteria
365	*Chlorobium limicola DSM 245*	Chlorobi	Bacteria
366	*Chlorobaculum parvum NCIB 8327*	Chlorobi	Bacteria
367	*Chlorobium tepidum TLS*	Chlorobi	Bacteria
368	*Chloroherpeton thalassium ATCC 35110*	Chlorobi	Bacteria
369	*Prosthecochloris aestuarii DSM 271*	Chlorobi	Bacteria
370	*Dehalococcoides sp. CBDB1*	Chloroflexi	Bacteria
371	*Dehalococcoides ethenogenes 195*	Chloroflexi	Bacteria
372	*Thermomicrobium roseum DSM 5159*	Chloroflexi	Bacteria
373	*Sphaerobacter thermophilus DSM 20745*	Chloroflexi	Bacteria
374	*Herpetosiphon aurantiacus ATCC 23779*	Chloroflexi	Bacteria
375	*Roseiflexus sp. RS-1*	Chloroflexi	Bacteria
376	*Roseiflexus castenholzii DSM 13941*	Chloroflexi	Bacteria
377	*Chloroflexus sp. Y-400-fl*	Chloroflexi	Bacteria
378	*Chloroflexus aggregans DSM 9485*	Chloroflexi	Bacteria
379	*Chloroflexus aurantiacus J-10-fl*	Chloroflexi	Bacteria
380	*Gloeobacter violaceus PCC 7421*	Cyanobacteria	Bacteria
381	*Acaryochloris marina MBIC11017*	Cyanobacteria	Bacteria
382	*Prochlorococcus marinus MIT 9313*	Cyanobacteria	Bacteria
383	*Nostoc punctiforme PCC 73102*	Cyanobacteria	Bacteria
384	*Nostoc sp. PCC 7120*	Cyanobacteria	Bacteria
385	*Anabaena variabilis ATCC 29413*	Cyanobacteria	Bacteria
386	*Trichodesmium erythraeum IMS101*	Cyanobacteria	Bacteria
387	*Thermosynechococcus elongatus BP-1*	Cyanobacteria	Bacteria
388	*cyanobacterium UCYN-A*	Cyanobacteria	Bacteria
389	*Cyanothece sp. ATCC 51142*	Cyanobacteria	Bacteria
390	*Synechocystis sp. PCC 6803*	Cyanobacteria	Bacteria
391	*Synechococcus elongatus PCC 6301*	Cyanobacteria	Bacteria
392	*Microcystis aeruginosa NIES-843*	Cyanobacteria	Bacteria
393	*Denitrovibrio acetiphilus DSM 12809*	Deferribacteres	Bacteria
394	*Deferribacter desulfuricans SSM1*	Deferribacteres	Bacteria
395	*Deinococcus deserti VCD115*	Deinococcus-Thermus	Bacteria
396	*Deinococcus geothermalis DSM 11300*	Deinococcus-Thermus	Bacteria
397	*Deinococcus radiodurans R1*	Deinococcus-Thermus	Bacteria
398	*Meiothermus ruber DSM 1279*	Deinococcus-Thermus	Bacteria
399	*Thermus thermophilus HB27*	Deinococcus-Thermus	Bacteria
400	*Dictyoglomus turgidum DSM 6724*	Dictyoglomi	Bacteria
401	*Dictyoglomus thermophilum H-6-12*	Dictyoglomi	Bacteria
402	*Elusimicrobium minutum Pei191*	Elusimicrobia	Bacteria
403	*uncultured Termite group 1 bacterium phylotype Rs-D17*	Elusimicrobia	Bacteria
404	*Fibrobacter succinogenes ssp. succinogenes S85*	Fibrobacteres	Bacteria
405	*Acidaminococcus fermentans DSM 20731*	Firmicutes	Bacteria
406	*Veillonella parvula DSM 2008*	Firmicutes	Bacteria
407	*Natranaerobius thermophilus JW/NM-WN-LF*	Firmicutes	Bacteria
408	*Symbiobacterium thermophilum IAM 14863*	Firmicutes	Bacteria
409	*Anaerococcus prevotii DSM 20548*	Firmicutes	Bacteria
410	*Finegoldia magna ATCC 29328*	Firmicutes	Bacteria
411	*Clostridiales genomosp. BVAB3 UPII9-5*	Firmicutes	Bacteria
412	*Candidatus Desulforudis audaxviator MP104C*	Firmicutes	Bacteria
413	*Pelotomaculum thermopropionicum SI*	Firmicutes	Bacteria
414	*Desulfitobacterium hafniense Y51*	Firmicutes	Bacteria
415	*Desulfotomaculum reducens MI-1*	Firmicutes	Bacteria
416	*Desulfotomaculum acetoxidans DSM 771*	Firmicutes	Bacteria
417	*Eubacterium rectale ATCC 33656*	Firmicutes	Bacteria
418	*Eubacterium eligens ATCC 27750*	Firmicutes	Bacteria
419	*Syntrophomonas wolfei ssp. wolfei Goettingen*	Firmicutes	Bacteria
420	*Heliobacterium modesticaldum Ice1*	Firmicutes	Bacteria
421	*Alkaliphilus oremlandii OhILAs*	Firmicutes	Bacteria
422	*Alkaliphilus metalliredigens QYMF*	Firmicutes	Bacteria
423	*Clostridium phytofermentans ISDg*	Firmicutes	Bacteria
424	*Clostridium novyi NT*	Firmicutes	Bacteria
425	*Clostridium kluyveri DSM 555*	Firmicutes	Bacteria
426	*Clostridium cellulolyticum H10*	Firmicutes	Bacteria
427	*Clostridium beijerinckii NCIMB 8052*	Firmicutes	Bacteria
428	*Clostridium thermocellum ATCC 27405*	Firmicutes	Bacteria
429	*Clostridium tetani E88*	Firmicutes	Bacteria
430	*Clostridium perfringens 13*	Firmicutes	Bacteria
431	*Clostridium difficile 630*	Firmicutes	Bacteria
432	*Clostridium botulinum A ATCC 3502*	Firmicutes	Bacteria
433	*Clostridium acetobutylicum ATCC 824*	Firmicutes	Bacteria
434	*Caldicellulosiruptor saccharolyticus DSM 8903*	Firmicutes	Bacteria
435	*Anaerocellum thermophilum DSM 6725*	Firmicutes	Bacteria
436	*Coprothermobacter proteolyticus DSM 5265*	Firmicutes	Bacteria
437	*Thermoanaerobacter tengcongensis MB4*	Firmicutes	Bacteria
438	*Carboxydothermus hydrogenoformans Z-2901*	Firmicutes	Bacteria
439	*Moorella thermoacetica ATCC 39073*	Firmicutes	Bacteria
440	*Ammonifex degensii KC4*	Firmicutes	Bacteria
441	*Thermoanaerobacter pseudethanolicus ATCC 33223*	Firmicutes	Bacteria
442	*Thermoanaerobacter sp. X514*	Firmicutes	Bacteria
443	*Thermoanaerobacter italicus Ab9*	Firmicutes	Bacteria
444	*Halothermothrix orenii H 168*	Firmicutes	Bacteria
445	*Enterococcus faecalis V583*	Firmicutes	Bacteria
446	*Oenococcus oeni PSU-1*	Firmicutes	Bacteria
447	*Leuconostoc citreum KM20*	Firmicutes	Bacteria
448	*Leuconostoc mesenteroides ssp. mesenteroides ATCC 8293*	Firmicutes	Bacteria
449	*Lactobacillus casei ATCC 334*	Firmicutes	Bacteria
450	*Lactobacillus crispatus ST1*	Firmicutes	Bacteria
451	*Lactobacillus rhamnosus GG*	Firmicutes	Bacteria
452	*Lactobacillus johnsonii NCC 533*	Firmicutes	Bacteria
453	*Lactobacillus salivarius UCC118*	Firmicutes	Bacteria
454	*Lactobacillus fermentum IFO 3956*	Firmicutes	Bacteria
455	*Lactobacillus sakei ssp. sakei 23K*	Firmicutes	Bacteria
456	*Lactobacillus reuteri DSM 20016*	Firmicutes	Bacteria
457	*Lactobacillus gasseri ATCC 33323*	Firmicutes	Bacteria
458	*Lactobacillus plantarum WCFS1*	Firmicutes	Bacteria
459	*Lactobacillus helveticus DPC 4571*	Firmicutes	Bacteria
460	*Lactobacillus delbrueckii ssp. bulgaricus ATCC 11842*	Firmicutes	Bacteria
461	*Lactobacillus brevis ATCC 367*	Firmicutes	Bacteria
462	*Lactobacillus acidophilus NCFM*	Firmicutes	Bacteria
463	*Pediococcus pentosaceus ATCC 25745*	Firmicutes	Bacteria
464	*Lactococcus lactis ssp. lactis Il1403*	Firmicutes	Bacteria
465	*Streptococcus gallolyticus UCN34*	Firmicutes	Bacteria
466	*Streptococcus equi ssp. zooepidemicus MGCS10565*	Firmicutes	Bacteria
467	*Streptococcus dysgalactiae ssp. equisimilis GGS_124*	Firmicutes	Bacteria
468	*Streptococcus mitis B6*	Firmicutes	Bacteria
469	*Streptococcus uberis 0140J*	Firmicutes	Bacteria
470	*Streptococcus pyogenes M1 GAS*	Firmicutes	Bacteria
471	*Streptococcus pneumoniae TIGR4*	Firmicutes	Bacteria
472	*Streptococcus agalactiae NEM316*	Firmicutes	Bacteria
473	*Streptococcus mutans UA159*	Firmicutes	Bacteria
474	*Streptococcus thermophilus LMG 18311*	Firmicutes	Bacteria
475	*Streptococcus suis 05ZYH33*	Firmicutes	Bacteria
476	*Streptococcus sanguinis SK36*	Firmicutes	Bacteria
477	*Streptococcus gordonii Challis subCH1*	Firmicutes	Bacteria
478	*Exiguobacterium sp. AT1b*	Firmicutes	Bacteria
479	*Exiguobacterium sibiricum 255-15*	Firmicutes	Bacteria
480	*Bacillus tusciae DSM 2912*	Firmicutes	Bacteria
481	*Alicyclobacillus acidocaldarius ssp. acidocaldarius DSM 446*	Firmicutes	Bacteria
482	*Brevibacillus brevis NBRC 100599*	Firmicutes	Bacteria
483	*Paenibacillus sp. JDR-2*	Firmicutes	Bacteria
484	*Listeria welshimeri ser. 6b SLCC5334*	Firmicutes	Bacteria
485	*Listeria innocua Clip11262*	Firmicutes	Bacteria
486	*Listeria seeligeri ser. 1/2b SLCC3954*	Firmicutes	Bacteria
487	*Listeria monocytogenes EGD-e*	Firmicutes	Bacteria
488	*Lysinibacillus sphaericus C3-41*	Firmicutes	Bacteria
489	*Oceanobacillus iheyensis HTE831*	Firmicutes	Bacteria
490	*Anoxybacillus flavithermus WK1*	Firmicutes	Bacteria
491	*Geobacillus sp. WCH70*	Firmicutes	Bacteria
492	*Geobacillus thermodenitrificans NG80-2*	Firmicutes	Bacteria
493	*Geobacillus kaustophilus HTA426*	Firmicutes	Bacteria
494	*Bacillus subtilis ssp. subtilis 168*	Firmicutes	Bacteria
495	*Bacillus licheniformis ATCC 14580*	Firmicutes	Bacteria
496	*Bacillus amyloliquefaciens FZB42*	Firmicutes	Bacteria
497	*Bacillus halodurans C-125*	Firmicutes	Bacteria
498	*Bacillus weihenstephanensis KBAB4*	Firmicutes	Bacteria
499	*Bacillus thuringiensis ser. konkukian 97-27*	Firmicutes	Bacteria
500	*Bacillus cereus ATCC 14579*	Firmicutes	Bacteria
501	*Bacillus anthracis Ames Ancestor*	Firmicutes	Bacteria
502	*Bacillus pseudofirmus OF4*	Firmicutes	Bacteria
503	*Bacillus clausii KSM-K16*	Firmicutes	Bacteria
504	*Bacillus pumilus SAFR-032*	Firmicutes	Bacteria
505	*Bacillus megaterium QM B1551*	Firmicutes	Bacteria
506	*Macrococcus caseolyticus JCSC5402*	Firmicutes	Bacteria
507	*Staphylococcus saprophyticus ssp. saprophyticus ATCC 15305*	Firmicutes	Bacteria
508	*Staphylococcus lugdunensis HKU09-01*	Firmicutes	Bacteria
509	*Staphylococcus haemolyticus JCSC1435*	Firmicutes	Bacteria
510	*Staphylococcus epidermidis RP62A*	Firmicutes	Bacteria
511	*Staphylococcus carnosus ssp. carnosus TM300*	Firmicutes	Bacteria
512	*Staphylococcus aureus ssp. aureus NCTC 8325*	Firmicutes	Bacteria
513	*Streptobacillus moniliformis DSM 12112*	Fusobacteria	Bacteria
514	*Sebaldella termitidis ATCC 33386*	Fusobacteria	Bacteria
515	*Leptotrichia buccalis C-1013-b*	Fusobacteria	Bacteria
516	*Fusobacterium nucleatum ssp. nucleatum ATCC 25586*	Fusobacteria	Bacteria
517	*Gemmatimonas aurantiaca T-27*	Gemmatimonadetes	Bacteria
518	*Thermodesulfovibrio yellowstonii DSM 11347*	Nitrospirae	Bacteria
519	*Rhodopirellula baltica SH 1*	Planctomycetes	Bacteria
520	*Pirellula staleyi DSM 6068*	Planctomycetes	Bacteria
521	*Nautilia profundicola AmH*	Proteobacteria	Bacteria
522	*Sulfurospirillum deleyianum DSM 6946*	Proteobacteria	Bacteria
523	*Arcobacter butzleri RM4018*	Proteobacteria	Bacteria
524	*Campylobacter hominis ATCC BAA-381*	Proteobacteria	Bacteria
525	*Campylobacter lari RM2100*	Proteobacteria	Bacteria
526	*Campylobacter curvus 525.92*	Proteobacteria	Bacteria
527	*Campylobacter concisus 13826*	Proteobacteria	Bacteria
528	*Campylobacter jejuni ssp. jejuni NCTC 11168*	Proteobacteria	Bacteria
529	*Campylobacter fetus ssp. fetus 82-40*	Proteobacteria	Bacteria
530	*Sulfurimonas denitrificans DSM 1251*	Proteobacteria	Bacteria
531	*Wolinella succinogenes DSM 1740*	Proteobacteria	Bacteria
532	*Helicobacter hepaticus ATCC 51449*	Proteobacteria	Bacteria
533	*Helicobacter mustelae 12198*	Proteobacteria	Bacteria
534	*Helicobacter acinonychis Sheeba*	Proteobacteria	Bacteria
535	*Helicobacter pylori 26695*	Proteobacteria	Bacteria
536	*Nitratiruptor sp. SB155-2*	Proteobacteria	Bacteria
537	*Sulfurovum sp. NBC37-1*	Proteobacteria	Bacteria
538	*Bdellovibrio bacteriovorus HD100*	Proteobacteria	Bacteria
539	*Syntrophus aciditrophicus SB*	Proteobacteria	Bacteria
540	*Syntrophobacter fumaroxidans MPOB*	Proteobacteria	Bacteria
541	*Desulfotalea psychrophila LSv54*	Proteobacteria	Bacteria
542	*Desulfatibacillum alkenivorans AK-01*	Proteobacteria	Bacteria
543	*Desulfobacterium autotrophicum HRM2*	Proteobacteria	Bacteria
544	*Desulfococcus oleovorans Hxd3*	Proteobacteria	Bacteria
545	*Desulfohalobium retbaense DSM 5692*	Proteobacteria	Bacteria
546	*Desulfomicrobium baculatum DSM 4028*	Proteobacteria	Bacteria
547	*Lawsonia intracellularis PHE/MN1-00*	Proteobacteria	Bacteria
548	*Desulfovibrio magneticus RS-1*	Proteobacteria	Bacteria
549	*Desulfovibrio vulgaris Hildenborough*	Proteobacteria	Bacteria
550	*Desulfovibrio salexigens DSM 2638*	Proteobacteria	Bacteria
551	*Desulfovibrio desulfuricans ssp. desulfuricans G20*	Proteobacteria	Bacteria
552	*Pelobacter propionicus DSM 2379*	Proteobacteria	Bacteria
553	*Pelobacter carbinolicus DSM 2380*	Proteobacteria	Bacteria
554	*Geobacter uraniireducens Rf4*	Proteobacteria	Bacteria
555	*Geobacter sp. FRC-32*	Proteobacteria	Bacteria
556	*Geobacter lovleyi SZ*	Proteobacteria	Bacteria
557	*Geobacter bemidjiensis Bem*	Proteobacteria	Bacteria
558	*Geobacter sulfurreducens PCA*	Proteobacteria	Bacteria
559	*Geobacter metallireducens GS-15*	Proteobacteria	Bacteria
560	*Haliangium ochraceum DSM 14365*	Proteobacteria	Bacteria
561	*Sorangium cellulosum So ce 56*	Proteobacteria	Bacteria
562	*Anaeromyxobacter sp. Fw109-5*	Proteobacteria	Bacteria
563	*Anaeromyxobacter dehalogenans 2CP-C*	Proteobacteria	Bacteria
564	*Myxococcus xanthus DK 1622*	Proteobacteria	Bacteria
565	*Magnetococcus sp. MC-1*	Proteobacteria	Bacteria
566	*Sideroxydans lithotrophicus ES-1*	Proteobacteria	Bacteria
567	*Aromatoleum aromaticum EbN1*	Proteobacteria	Bacteria
568	*Dechloromonas aromatica RCB*	Proteobacteria	Bacteria
569	*Thauera sp. MZ1T*	Proteobacteria	Bacteria
570	*Laribacter hongkongensis HLHK9*	Proteobacteria	Bacteria
571	*Chromobacterium violaceum ATCC 12472*	Proteobacteria	Bacteria
572	*Neisseria meningitidis Z2491*	Proteobacteria	Bacteria
573	*Neisseria gonorrhoeae FA 1090*	Proteobacteria	Bacteria
574	*Methylotenera mobilis JLW8*	Proteobacteria	Bacteria
575	*Methylovorus sp. SIP3-4*	Proteobacteria	Bacteria
576	*Methylobacillus flagellatus KT*	Proteobacteria	Bacteria
577	*Thiobacillus denitrificans ATCC 25259*	Proteobacteria	Bacteria
578	*Candidatus Accumulibacter phosphatis clade IIA UW-1*	Proteobacteria	Bacteria
579	*Methylibium petroleiphilum PM1*	Proteobacteria	Bacteria
580	*Leptothrix cholodnii SP-6*	Proteobacteria	Bacteria
581	*Ralstonia eutropha JMP134*	Proteobacteria	Bacteria
582	*Cupriavidus taiwanensis*	Proteobacteria	Bacteria
583	*Cupriavidus metallidurans CH34*	Proteobacteria	Bacteria
584	*Ralstonia pickettii 12J*	Proteobacteria	Bacteria
585	*Ralstonia solanacearum GMI1000*	Proteobacteria	Bacteria
586	*Polynucleobacter necessarius ssp. asymbioticus QLW-P1DMWA-1*	Proteobacteria	Bacteria
587	*Burkholderia phytofirmans PsJN*	Proteobacteria	Bacteria
588	*Burkholderia phymatum STM815*	Proteobacteria	Bacteria
589	*Burkholderia thailandensis E264*	Proteobacteria	Bacteria
590	*Burkholderia pseudomallei K96243*	Proteobacteria	Bacteria
591	*Burkholderia mallei ATCC 23344*	Proteobacteria	Bacteria
592	*Burkholderia sp. 383*	Proteobacteria	Bacteria
593	*Burkholderia ambifaria AMMD*	Proteobacteria	Bacteria
594	*Burkholderia cenocepacia AU 1054*	Proteobacteria	Bacteria
595	*Burkholderia multivorans ATCC 17616*	Proteobacteria	Bacteria
596	*Burkholderia vietnamiensis G4*	Proteobacteria	Bacteria
597	*Burkholderia xenovorans LB400*	Proteobacteria	Bacteria
598	*Burkholderia glumae BGR1*	Proteobacteria	Bacteria
599	*Rhodoferax ferrireducens T118*	Proteobacteria	Bacteria
600	*Verminephrobacter eiseniae EF01-2*	Proteobacteria	Bacteria
601	*Delftia acidovorans SPH-1*	Proteobacteria	Bacteria
602	*Polaromonas sp. JS666*	Proteobacteria	Bacteria
603	*Polaromonas naphthalenivorans CJ2*	Proteobacteria	Bacteria
604	*Variovorax paradoxus S110*	Proteobacteria	Bacteria
605	*Acidovorax ebreus TPSY*	Proteobacteria	Bacteria
606	*Acidovorax sp. JS42*	Proteobacteria	Bacteria
607	*Acidovorax citrulli AAC00-1*	Proteobacteria	Bacteria
608	*Herminiimonas arsenicoxydans*	Proteobacteria	Bacteria
609	*Janthinobacterium sp. Marseille*	Proteobacteria	Bacteria
610	*Bordetella petrii DSM 12804*	Proteobacteria	Bacteria
611	*Bordetella avium 197N*	Proteobacteria	Bacteria
612	*Bordetella pertussis Tohama I*	Proteobacteria	Bacteria
613	*Bordetella parapertussis 12822*	Proteobacteria	Bacteria
614	*Bordetella bronchiseptica RB50*	Proteobacteria	Bacteria
615	*Nitrosospira multiformis ATCC 25196*	Proteobacteria	Bacteria
616	*Nitrosomonas eutropha C91*	Proteobacteria	Bacteria
617	*Nitrosomonas europaea ATCC 19718*	Proteobacteria	Bacteria
618	*Caulobacter sp. K31*	Proteobacteria	Bacteria
619	*Caulobacter crescentus CB15*	Proteobacteria	Bacteria
620	*Caulobacter segnis ATCC 21756*	Proteobacteria	Bacteria
621	*Phenylobacterium zucineum HLK1*	Proteobacteria	Bacteria
622	*Erythrobacter litoralis HTCC2594*	Proteobacteria	Bacteria
623	*Sphingopyxis alaskensis RB2256*	Proteobacteria	Bacteria
624	*Novosphingobium aromaticivorans DSM 12444*	Proteobacteria	Bacteria
625	*Sphingobium japonicum UT26S*	Proteobacteria	Bacteria
626	*Sphingomonas wittichii RW1*	Proteobacteria	Bacteria
627	*Zymomonas mobilis ssp. mobilis ZM4*	Proteobacteria	Bacteria
628	*Maricaulis maris MCS10*	Proteobacteria	Bacteria
629	*Hirschia baltica ATCC 49814*	Proteobacteria	Bacteria
630	*Hyphomonas neptunium ATCC 15444*	Proteobacteria	Bacteria
631	*Dinoroseobacter shibae DFL 12*	Proteobacteria	Bacteria
632	*Jannaschia sp. CCS1*	Proteobacteria	Bacteria
633	*Ruegeria sp. TM1040*	Proteobacteria	Bacteria
634	*Ruegeria pomeroyi DSS-3*	Proteobacteria	Bacteria
635	*Roseobacter denitrificans OCh 114*	Proteobacteria	Bacteria
636	*Rhodobacter sphaeroides 2.4.1*	Proteobacteria	Bacteria
637	*Rhodobacter capsulatus SB 1003*	Proteobacteria	Bacteria
638	*Paracoccus denitrificans PD1222*	Proteobacteria	Bacteria
639	*Magnetospirillum magneticum AMB-1*	Proteobacteria	Bacteria
640	*Rhodospirillum centenum SW*	Proteobacteria	Bacteria
641	*Rhodospirillum rubrum ATCC 11170*	Proteobacteria	Bacteria
642	*Azospirillum sp. B510*	Proteobacteria	Bacteria
643	*Granulibacter bethesdensis CGDNIH1*	Proteobacteria	Bacteria
644	*Gluconacetobacter diazotrophicus PAl 5*	Proteobacteria	Bacteria
645	*Gluconobacter oxydans 621H*	Proteobacteria	Bacteria
646	*Acetobacter pasteurianus IFO 3283-01*	Proteobacteria	Bacteria
647	*Candidatus Puniceispirillum marinum IMCC1322*	Proteobacteria	Bacteria
648	*Candidatus Pelagibacter ubique HTCC1062*	Proteobacteria	Bacteria
649	*Neorickettsia sennetsu Miyayama*	Proteobacteria	Bacteria
650	*Neorickettsia risticii Illinois*	Proteobacteria	Bacteria
651	*Wolbachia endosymbiont of Culex_quinquefasciatus Pel*	Proteobacteria	Bacteria
652	*Wolbachia endosymbiont of Drosophila melanogaster*	Proteobacteria	Bacteria
653	*Wolbachia endosymbiont TRS of Brugia malayi*	Proteobacteria	Bacteria
654	*Wolbachia sp. wRi*	Proteobacteria	Bacteria
655	*Ehrlichia chaffeensis Arkansas*	Proteobacteria	Bacteria
656	*Ehrlichia canis Jake*	Proteobacteria	Bacteria
657	*Ehrlichia ruminantium Welgevonden*	Proteobacteria	Bacteria
658	*Anaplasma phagocytophilum HZ*	Proteobacteria	Bacteria
659	*Anaplasma marginale St. Maries*	Proteobacteria	Bacteria
660	*Anaplasma centrale Israel*	Proteobacteria	Bacteria
661	*Orientia tsutsugamushi Boryong*	Proteobacteria	Bacteria
662	*Rickettsia bellii RML369-C*	Proteobacteria	Bacteria
663	*Rickettsia canadensis McKiel*	Proteobacteria	Bacteria
664	*Rickettsia typhi Wilmington*	Proteobacteria	Bacteria
665	*Rickettsia prowazekii Madrid E*	Proteobacteria	Bacteria
666	*Rickettsia peacockii Rustic*	Proteobacteria	Bacteria
667	*Rickettsia felis URRWXCal2*	Proteobacteria	Bacteria
668	*Rickettsia massiliae MTU5*	Proteobacteria	Bacteria
669	*Rickettsia africae ESF-5*	Proteobacteria	Bacteria
670	*Rickettsia akari Hartford*	Proteobacteria	Bacteria
671	*Rickettsia rickettsii Sheila Smith*	Proteobacteria	Bacteria
672	*Rickettsia conorii Malish 7*	Proteobacteria	Bacteria
673	*Xanthobacter autotrophicus Py2*	Proteobacteria	Bacteria
674	*Azorhizobium caulinodans ORS 571*	Proteobacteria	Bacteria
675	*Methylobacterium chloromethanicum CM4*	Proteobacteria	Bacteria
676	*Methylobacterium extorquens PA1*	Proteobacteria	Bacteria
677	*Methylobacterium sp. 4-46*	Proteobacteria	Bacteria
678	*Methylobacterium populi BJ001*	Proteobacteria	Bacteria
679	*Methylobacterium nodulans ORS 2060*	Proteobacteria	Bacteria
680	*Methylobacterium radiotolerans JCM 2831*	Proteobacteria	Bacteria
681	*Candidatus Hodgkinia cicadicola Dsem*	Proteobacteria	Bacteria
682	*Ochrobactrum anthropi ATCC 49188*	Proteobacteria	Bacteria
683	*Brucella microti CCM 4915*	Proteobacteria	Bacteria
684	*Brucella canis ATCC 23365*	Proteobacteria	Bacteria
685	*Brucella suis 1330*	Proteobacteria	Bacteria
686	*Brucella melitensis bv. 1 16M*	Proteobacteria	Bacteria
687	*Brucella ovis ATCC 25840*	Proteobacteria	Bacteria
688	*Brucella abortus bv. 1 9-941*	Proteobacteria	Bacteria
689	*Rhizobium sp. NGR234*	Proteobacteria	Bacteria
690	*Sinorhizobium medicae WSM419*	Proteobacteria	Bacteria
691	*Sinorhizobium meliloti 1021*	Proteobacteria	Bacteria
692	*Rhizobium etli CFN 42*	Proteobacteria	Bacteria
693	*Rhizobium leguminosarum bv. viciae 3841*	Proteobacteria	Bacteria
694	*Agrobacterium vitis S4*	Proteobacteria	Bacteria
695	*Agrobacterium radiobacter K84*	Proteobacteria	Bacteria
696	*Agrobacterium tumefaciens C58*	Proteobacteria	Bacteria
697	*Candidatus Liberibacter asiaticus psy62*	Proteobacteria	Bacteria
698	*Chelativorans sp. BNC1*	Proteobacteria	Bacteria
699	*Parvibaculum lavamentivorans DS-1*	Proteobacteria	Bacteria
700	*Mesorhizobium loti MAFF303099*	Proteobacteria	Bacteria
701	*Methylocella silvestris BL2*	Proteobacteria	Bacteria
702	*Beijerinckia indica ssp. indica ATCC 9039*	Proteobacteria	Bacteria
703	*Oligotropha carboxidovorans OM5*	Proteobacteria	Bacteria
704	*Rhodopseudomonas palustris CGA009*	Proteobacteria	Bacteria
705	*Nitrobacter winogradskyi Nb-255*	Proteobacteria	Bacteria
706	*Nitrobacter hamburgensis X14*	Proteobacteria	Bacteria
707	*Bradyrhizobium sp. ORS278*	Proteobacteria	Bacteria
708	*Bradyrhizobium japonicum USDA 110*	Proteobacteria	Bacteria
709	*Bartonella tribocorum CIP 105476*	Proteobacteria	Bacteria
710	*Bartonella henselae Houston-1*	Proteobacteria	Bacteria
711	*Bartonella grahamii as4aup*	Proteobacteria	Bacteria
712	*Bartonella quintana Toulouse*	Proteobacteria	Bacteria
713	*Bartonella bacilliformis KC583*	Proteobacteria	Bacteria
714	*Acidithiobacillus ferrooxidans ATCC 23270*	Proteobacteria	Bacteria
715	*Mannheimia succiniciproducens MBEL55E*	Proteobacteria	Bacteria
716	*Aggregatibacter aphrophilus NJ8700*	Proteobacteria	Bacteria
717	*Aggregatibacter actinomycetemcomitans D11S-1*	Proteobacteria	Bacteria
718	*Haemophilus somnus 129PT*	Proteobacteria	Bacteria
719	*Pasteurella multocida ssp. multocida Pm70*	Proteobacteria	Bacteria
720	*Haemophilus parasuis SH0165*	Proteobacteria	Bacteria
721	*Haemophilus ducreyi 35000HP*	Proteobacteria	Bacteria
722	*Haemophilus influenzae Rd KW20*	Proteobacteria	Bacteria
723	*Actinobacillus succinogenes 130Z*	Proteobacteria	Bacteria
724	*Actinobacillus pleuropneumoniae L20*	Proteobacteria	Bacteria
725	*Tolumonas auensis DSM 9187*	Proteobacteria	Bacteria
726	*Aeromonas salmonicida ssp. salmonicida A449*	Proteobacteria	Bacteria
727	*Aeromonas hydrophila ssp. hydrophila ATCC 7966*	Proteobacteria	Bacteria
728	*Aliivibrio salmonicida LFI1238*	Proteobacteria	Bacteria
729	*Vibrio fischeri ES114*	Proteobacteria	Bacteria
730	*Vibrio parahaemolyticus RIMD 2210633*	Proteobacteria	Bacteria
731	*Vibrio harveyi ATCC BAA-1116*	Proteobacteria	Bacteria
732	*Vibrio sp. Ex25*	Proteobacteria	Bacteria
733	*Vibrio splendidus LGP32*	Proteobacteria	Bacteria
734	*Vibrio vulnificus YJ016*	Proteobacteria	Bacteria
735	*Vibrio cholerae O1 biov. El Tor N16961*	Proteobacteria	Bacteria
736	*Photobacterium profundum SS9*	Proteobacteria	Bacteria
737	*Psychromonas ingrahamii 37*	Proteobacteria	Bacteria
738	*Idiomarina loihiensis L2TR*	Proteobacteria	Bacteria
739	*Shewanella piezotolerans WP3*	Proteobacteria	Bacteria
740	*Shewanella loihica PV-4*	Proteobacteria	Bacteria
741	*Shewanella halifaxensis HAW-EB4*	Proteobacteria	Bacteria
742	*Shewanella sediminis HAW-EB3*	Proteobacteria	Bacteria
743	*Shewanella denitrificans OS217*	Proteobacteria	Bacteria
744	*Shewanella pealeana ATCC 700345*	Proteobacteria	Bacteria
745	*Shewanella oneidensis MR-1*	Proteobacteria	Bacteria
746	*Shewanella baltica OS155*	Proteobacteria	Bacteria
747	*Shewanella woodyi ATCC 51908*	Proteobacteria	Bacteria
748	*Shewanella sp. MR-7*	Proteobacteria	Bacteria
749	*Shewanella amazonensis SB2B*	Proteobacteria	Bacteria
750	*Shewanella violacea DSS12*	Proteobacteria	Bacteria
751	*Shewanella frigidimarina NCIMB 400*	Proteobacteria	Bacteria
752	*Shewanella putrefaciens CN-32*	Proteobacteria	Bacteria
753	*Colwellia psychrerythraea 34H*	Proteobacteria	Bacteria
754	*Pseudoalteromonas atlantica T6c*	Proteobacteria	Bacteria
755	*Pseudoalteromonas haloplanktis TAC125*	Proteobacteria	Bacteria
756	*Teredinibacter turnerae T7901*	Proteobacteria	Bacteria
757	*Saccharophagus degradans 2-40*	Proteobacteria	Bacteria
758	*Marinobacter aquaeolei VT8*	Proteobacteria	Bacteria
759	*Alteromonas macleodii Deep ecotype*	Proteobacteria	Bacteria
760	*Hahella chejuensis KCTC 2396*	Proteobacteria	Bacteria
761	*Kangiella koreensis DSM 16069*	Proteobacteria	Bacteria
762	*Alcanivorax borkumensis SK2*	Proteobacteria	Bacteria
763	*Marinomonas sp. MWYL1*	Proteobacteria	Bacteria
764	*Chromohalobacter salexigens DSM 3043*	Proteobacteria	Bacteria
765	*Methylococcus capsulatus Bath*	Proteobacteria	Bacteria
766	*Dichelobacter nodosus VCS1703A*	Proteobacteria	Bacteria
767	*Stenotrophomonas maltophilia R551-3*	Proteobacteria	Bacteria
768	*Xylella fastidiosa 9a5c*	Proteobacteria	Bacteria
769	*Xanthomonas axonopodis pv. citri 306*	Proteobacteria	Bacteria
770	*Xanthomonas albilineans*	Proteobacteria	Bacteria
771	*Xanthomonas oryzae pv. oryzae KACC10331*	Proteobacteria	Bacteria
772	*Xanthomonas campestris pv. campestris ATCC 33913*	Proteobacteria	Bacteria
773	*Halothiobacillus neapolitanus c2*	Proteobacteria	Bacteria
774	*Alkalilimnicola ehrlichii MLHE-1*	Proteobacteria	Bacteria
775	*Thioalkalivibrio sp. HL-EbGR7*	Proteobacteria	Bacteria
776	*Halorhodospira halophila SL1*	Proteobacteria	Bacteria
777	*Allochromatium vinosum DSM 180*	Proteobacteria	Bacteria
778	*Nitrosococcus halophilus Nc4*	Proteobacteria	Bacteria
779	*Nitrosococcus oceani ATCC 19707*	Proteobacteria	Bacteria
780	*Coxiella burnetii RSA 493*	Proteobacteria	Bacteria
781	*Legionella longbeachae NSW150*	Proteobacteria	Bacteria
782	*Legionella pneumophila ssp. pneumophila Philadelphia 1*	Proteobacteria	Bacteria
783	*Baumannia cicadellinicola Hc*	Proteobacteria	Bacteria
784	*Candidatus Carsonella ruddii PV*	Proteobacteria	Bacteria
785	*Candidatus Vesicomyosocius okutanii HA*	Proteobacteria	Bacteria
786	*Candidatus Ruthia magnifica Cm*	Proteobacteria	Bacteria
787	*Cronobacter turicensis z3032*	Proteobacteria	Bacteria
788	*Cronobacter sakazakii ATCC BAA-894*	Proteobacteria	Bacteria
789	*Candidatus Riesia pediculicola USDA*	Proteobacteria	Bacteria
790	*Dickeya zeae Ech1591*	Proteobacteria	Bacteria
791	*Dickeya dadantii Ech703*	Proteobacteria	Bacteria
792	*Candidatus Hamiltonella defensa 5AT*	Proteobacteria	Bacteria
793	*Candidatus Blochmannia floridanus*	Proteobacteria	Bacteria
794	*Pectobacterium wasabiae WPP163*	Proteobacteria	Bacteria
795	*Pectobacterium atrosepticum SCRI1043*	Proteobacteria	Bacteria
796	*Pectobacterium carotovorum ssp. carotovorum PC1*	Proteobacteria	Bacteria
797	*Sodalis glossinidius morsitans*	Proteobacteria	Bacteria
798	*Pantoea ananatis LMG 20103*	Proteobacteria	Bacteria
799	*Wigglesworthia glossinidia*	Proteobacteria	Bacteria
800	*Buchnera aphidicola APS*	Proteobacteria	Bacteria
801	*Photorhabdus asymbiotica*	Proteobacteria	Bacteria
802	*Photorhabdus luminescens ssp. laumondii TTO1*	Proteobacteria	Bacteria
803	*Edwardsiella ictaluri 93-146*	Proteobacteria	Bacteria
804	*Edwardsiella tarda EIB202*	Proteobacteria	Bacteria
805	*Yersinia pseudotuberculosis IP 32953*	Proteobacteria	Bacteria
806	*Yersinia pestis CO92*	Proteobacteria	Bacteria
807	*Yersinia enterocolitica ssp. enterocolitica 8081*	Proteobacteria	Bacteria
808	*Xenorhabdus bovienii SS-2004*	Proteobacteria	Bacteria
809	*Shigella sonnei Ss046*	Proteobacteria	Bacteria
810	*Shigella flexneri 2a 2457T*	Proteobacteria	Bacteria
811	*Shigella dysenteriae Sd197*	Proteobacteria	Bacteria
812	*Shigella boydii Sb227*	Proteobacteria	Bacteria
813	*Serratia proteamaculans 568*	Proteobacteria	Bacteria
814	*Salmonella enterica ssp. enterica ser. Typhimurium LT2*	Proteobacteria	Bacteria
815	*Proteus mirabilis HI4320*	Proteobacteria	Bacteria
816	*Klebsiella variicola At-22*	Proteobacteria	Bacteria
817	*Klebsiella pneumoniae ssp. pneumoniae MGH 78578*	Proteobacteria	Bacteria
818	*Escherichia fergusonii ATCC 35469*	Proteobacteria	Bacteria
819	*Escherichia coli K-12 subMG1655*	Proteobacteria	Bacteria
820	*Erwinia tasmaniensis Et1/99*	Proteobacteria	Bacteria
821	*Erwinia pyrifoliae Ep1/96*	Proteobacteria	Bacteria
822	*Erwinia amylovora ATCC 49946*	Proteobacteria	Bacteria
823	*Enterobacter sp. 638*	Proteobacteria	Bacteria
824	*Citrobacter rodentium ICC168*	Proteobacteria	Bacteria
825	*Citrobacter koseri ATCC BAA-895*	Proteobacteria	Bacteria
826	*Azotobacter vinelandii DJ*	Proteobacteria	Bacteria
827	*Pseudomonas entomophila L48*	Proteobacteria	Bacteria
828	*Pseudomonas syringae pv. tomato DC3000*	Proteobacteria	Bacteria
829	*Pseudomonas stutzeri A1501*	Proteobacteria	Bacteria
830	*Pseudomonas putida KT2440*	Proteobacteria	Bacteria
831	*Pseudomonas fluorescens Pf-5*	Proteobacteria	Bacteria
832	*Pseudomonas mendocina ymp*	Proteobacteria	Bacteria
833	*Pseudomonas aeruginosa PAO1*	Proteobacteria	Bacteria
834	*Cellvibrio japonicus Ueda107*	Proteobacteria	Bacteria
835	*Psychrobacter sp. PRwf-1*	Proteobacteria	Bacteria
836	*Psychrobacter arcticus 273-4*	Proteobacteria	Bacteria
837	*Psychrobacter cryohalolentis K5*	Proteobacteria	Bacteria
838	*Acinetobacter baumannii ATCC 17978*	Proteobacteria	Bacteria
839	*Acinetobacter sp. ADP1*	Proteobacteria	Bacteria
840	*Thiomicrospira crunogena XCL-2*	Proteobacteria	Bacteria
841	*Francisella philomiragia ssp. philomiragia ATCC 25017*	Proteobacteria	Bacteria
842	*Francisella tularensis ssp. tularensis SCHU S4*	Proteobacteria	Bacteria
843	*Brachyspira hyodysenteriae WA1*	Spirochaetes	Bacteria
844	*Leptospira borgpetersenii ser. Hardjo-bovis L550*	Spirochaetes	Bacteria
845	*Leptospira interrogans ser. Lai 56601*	Spirochaetes	Bacteria
846	*Leptospira biflexa ser. Patoc Patoc 1 (Paris)*	Spirochaetes	Bacteria
847	*Treponema pallidum ssp. pallidum Nichols*	Spirochaetes	Bacteria
848	*Treponema denticola ATCC 35405*	Spirochaetes	Bacteria
849	*Borrelia garinii PBi*	Spirochaetes	Bacteria
850	*Borrelia afzelii PKo*	Spirochaetes	Bacteria
851	*Borrelia burgdorferi B31*	Spirochaetes	Bacteria
852	*Borrelia recurrentis A1*	Spirochaetes	Bacteria
853	*Borrelia duttonii Ly*	Spirochaetes	Bacteria
854	*Borrelia turicatae 91E135*	Spirochaetes	Bacteria
855	*Borrelia hermsii DAH*	Spirochaetes	Bacteria
856	*Aminobacterium colombiense DSM 12261*	Synergistetes	Bacteria
857	*Thermanaerovibrio acidaminovorans DSM 6589*	Synergistetes	Bacteria
858	*Candidatus Phytoplasma mali*	Tenericutes	Bacteria
859	*Aster yellows witches-broom phytoplasma AYWB*	Tenericutes	Bacteria
860	*Onion yellows phytoplasma OY-M*	Tenericutes	Bacteria
861	*Acholeplasma laidlawii PG-8A*	Tenericutes	Bacteria
862	*Mesoplasma florum L1*	Tenericutes	Bacteria
863	*Ureaplasma parvum ser. 3 ATCC 700970*	Tenericutes	Bacteria
864	*Ureaplasma urealyticum ser. 10 ATCC 33699*	Tenericutes	Bacteria
865	*Mycoplasma mycoides ssp. mycoides SC PG1*	Tenericutes	Bacteria
866	*Mycoplasma capricolum ssp. capricolum ATCC 27343*	Tenericutes	Bacteria
867	*Mycoplasma crocodyli MP145*	Tenericutes	Bacteria
868	*Mycoplasma conjunctivae HRC/581*	Tenericutes	Bacteria
869	*Mycoplasma penetrans HF-2*	Tenericutes	Bacteria
870	*Mycoplasma mobile 163K*	Tenericutes	Bacteria
871	*Mycoplasma arthritidis 158L3-1*	Tenericutes	Bacteria
872	*Mycoplasma agalactiae PG2*	Tenericutes	Bacteria
873	*Mycoplasma synoviae 53*	Tenericutes	Bacteria
874	*Mycoplasma pulmonis UAB CTIP*	Tenericutes	Bacteria
875	*Mycoplasma pneumoniae M129*	Tenericutes	Bacteria
876	*Mycoplasma hyopneumoniae 232*	Tenericutes	Bacteria
877	*Mycoplasma hominis*	Tenericutes	Bacteria
878	*Mycoplasma genitalium G37*	Tenericutes	Bacteria
879	*Mycoplasma gallisepticum R(low)*	Tenericutes	Bacteria
880	*Kosmotoga olearia TBF 19.5.1*	Thermotogae	Bacteria
881	*Petrotoga mobilis SJ95*	Thermotogae	Bacteria
882	*Fervidobacterium nodosum Rt17-B1*	Thermotogae	Bacteria
883	*Thermosipho melanesiensis BI429*	Thermotogae	Bacteria
884	*Thermosipho africanus TCF52B*	Thermotogae	Bacteria
885	*Thermotoga lettingae TMO*	Thermotogae	Bacteria
886	*Thermotoga sp. RQ2*	Thermotogae	Bacteria
887	*Thermotoga naphthophila RKU-10*	Thermotogae	Bacteria
888	*Thermotoga petrophila RKU-1*	Thermotogae	Bacteria
889	*Thermotoga neapolitana DSM 4359*	Thermotogae	Bacteria
890	*Thermotoga maritima MSB8*	Thermotogae	Bacteria
891	*Coraliomargarita akajimensis DSM 45221*	Verrucomicrobia	Bacteria
892	*Opitutus terrae PB90-1*	Verrucomicrobia	Bacteria
893	*Methylacidiphilum infernorum V4*	Verrucomicrobia	Bacteria
894	*Akkermansia muciniphila ATCC BAA-835*	Verrucomicrobia	Bacteria
895	*Thermobaculum terrenum ATCC BAA-798*		Bacteria
896	*Hyperthermus butylicus DSM 5456*	Crenarchaeota	Archaea
897	*Aeropyrum pernix K1*	Crenarchaeota	Archaea
898	*Ignicoccus hospitalis KIN4/I*	Crenarchaeota	Archaea
899	*Staphylothermus marinus F1*	Crenarchaeota	Archaea
900	*Desulfurococcus kamchatkensis 1221n*	Crenarchaeota	Archaea
901	*Metallosphaera sedula DSM 5348*	Crenarchaeota	Archaea
902	*Sulfolobus tokodaii 7*	Crenarchaeota	Archaea
903	*Sulfolobus islandicus Y.N.15.51*	Crenarchaeota	Archaea
904	*Sulfolobus solfataricus P2*	Crenarchaeota	Archaea
905	*Sulfolobus acidocaldarius DSM 639*	Crenarchaeota	Archaea
906	*Thermofilum pendens Hrk 5*	Crenarchaeota	Archaea
907	*Caldivirga maquilingensis IC-167*	Crenarchaeota	Archaea
908	*Pyrobaculum calidifontis JCM 11548*	Crenarchaeota	Archaea
909	*Pyrobaculum arsenaticum DSM 13514*	Crenarchaeota	Archaea
910	*Pyrobaculum aerophilum IM2*	Crenarchaeota	Archaea
911	*Pyrobaculum islandicum DSM 4184*	Crenarchaeota	Archaea
912	*Thermoproteus neutrophilus V24Sta*	Crenarchaeota	Archaea
913	*Methanocella paludicola SANAE*	Euryarchaeota	Archaea
914	*Methanosaeta thermophila PT*	Euryarchaeota	Archaea
915	*Methanococcoides burtonii DSM 6242*	Euryarchaeota	Archaea
916	*Methanosarcina acetivorans C2A*	Euryarchaeota	Archaea
917	*Methanosarcina mazei Go1*	Euryarchaeota	Archaea
918	*Methanosarcina barkeri Fusaro*	Euryarchaeota	Archaea
919	*Methanohalophilus mahii DSM 5219*	Euryarchaeota	Archaea
920	*Methanosphaerula palustris E1-9c*	Euryarchaeota	Archaea
921	*Candidatus Methanoregula boonei 6A8*	Euryarchaeota	Archaea
922	*Methanospirillum hungatei JF-1*	Euryarchaeota	Archaea
923	*Methanocorpusculum labreanum Z*	Euryarchaeota	Archaea
924	*Methanoculleus marisnigri JR1*	Euryarchaeota	Archaea
925	*Methanopyrus kandleri AV19*	Euryarchaeota	Archaea
926	*Ferroglobus placidus DSM 10642*	Euryarchaeota	Archaea
927	*Archaeoglobus profundus DSM 5631*	Euryarchaeota	Archaea
928	*Archaeoglobus fulgidus DSM 4304*	Euryarchaeota	Archaea
929	*Thermococcus onnurineus NA1*	Euryarchaeota	Archaea
930	*Thermococcus kodakarensis KOD1*	Euryarchaeota	Archaea
931	*Thermococcus gammatolerans EJ3*	Euryarchaeota	Archaea
932	*Thermococcus sibiricus MM 739*	Euryarchaeota	Archaea
933	*Pyrococcus horikoshii OT3*	Euryarchaeota	Archaea
934	*Pyrococcus abyssi GE5*	Euryarchaeota	Archaea
935	*Pyrococcus furiosus DSM 3638*	Euryarchaeota	Archaea
936	*Thermoplasma volcanium GSS1*	Euryarchaeota	Archaea
937	*Thermoplasma acidophilum DSM 1728*	Euryarchaeota	Archaea
938	*Picrophilus torridus DSM 9790*	Euryarchaeota	Archaea
939	*Haloquadratum walsbyi DSM 16790*	Euryarchaeota	Archaea
940	*Halomicrobium mukohataei DSM 12286*	Euryarchaeota	Archaea
941	*Halorhabdus utahensis DSM 12940*	Euryarchaeota	Archaea
942	*Haloterrigena turkmenica DSM 5511*	Euryarchaeota	Archaea
943	*Natronomonas pharaonis DSM 2160*	Euryarchaeota	Archaea
944	*Natrialba magadii ATCC 43099*	Euryarchaeota	Archaea
945	*Halorubrum lacusprofundi ATCC 49239*	Euryarchaeota	Archaea
946	*Haloferax volcanii DS2*	Euryarchaeota	Archaea
947	*Halobacterium salinarum R1*	Euryarchaeota	Archaea
948	*Halobacterium sp. NRC-1*	Euryarchaeota	Archaea
949	*Haloarcula marismortui ATCC 43049*	Euryarchaeota	Archaea
950	*Methanocaldococcus sp. FS406-22*	Euryarchaeota	Archaea
951	*Methanocaldococcus fervens AG86*	Euryarchaeota	Archaea
952	*Methanocaldococcus vulcanius M7*	Euryarchaeota	Archaea
953	*Methanocaldococcus jannaschii DSM 2661*	Euryarchaeota	Archaea
954	*Methanococcus aeolicus Nankai-3*	Euryarchaeota	Archaea
955	*Methanococcus maripaludis S2*	Euryarchaeota	Archaea
956	*Methanococcus vannielii SB*	Euryarchaeota	Archaea
957	*Methanothermobacter thermautotrophicus Delta H*	Euryarchaeota	Archaea
958	*Methanosphaera stadtmanae DSM 3091*	Euryarchaeota	Archaea
959	*Methanobrevibacter ruminantium M1*	Euryarchaeota	Archaea
960	*Methanobrevibacter smithii ATCC 35061*	Euryarchaeota	Archaea
961	*uncultured methanogenic archaeon RC-I*	Euryarchaeota	Archaea
962	*Aciduliprofundum boonei T469*	Euryarchaeota	Archaea
963	*Candidatus Korarchaeum cryptofilum OPF8*	Korarchaeota	Archaea
964	*Nanoarchaeum equitans Kin4-M*	Nanoarchaeota	Archaea
965	*Nitrosopumilus maritimus SCM1*	Thaumarchaeota	Archaea
